# Seasonal variation in egg nutrient composition under a pasture-based layer hen system: Implications for sustainable agriculture

**DOI:** 10.1371/journal.pone.0332411

**Published:** 2025-09-25

**Authors:** Rachel Van Duinen, Selin Sergin, Shreya Chavva, Julianna K. Adams, Chad A. Bitler, Jenifer I. Fenton

**Affiliations:** 1 Department of Food Science and Human Nutrition, Michigan State University, East Lansing, Michigan, United States of America; 2 Greenacres Foundation, Inc., Cincinnati, Ohio, United States of America; Ain Shams University Faculty of Agriculture, EGYPT

## Abstract

Sustainability in poultry production emphasizes systems that promote environmental health, animal welfare, and the potential to produce a more nutrient dense product. Pasture based poultry systems align with these sustainability goals by supporting soil fertility, biodiversity, and more natural behaviors. Access to pasture allows chickens to consume a diverse range of plants and insects, potentially enhancing the nutritional value of their eggs. However, environmental variability across the grazing season may influence egg nutrient profiles, impacting both nutritional quality and system resilience. This study evaluated how seasonal changes in forage quality, soil composition, and climate affect the nutrient profile of eggs produced under a regenerative, pasture-based system in Southern Ohio. Monthly collections of forage (n = 3) and eggs (n = 24, pooled into 12 replicates) occurred from May to December. Fatty acid composition was assessed using gas chromatography-mass spectrometry, while carotenoid and phenolic levels were measured colorimetrically. Vitamin and mineral content were analyzed through liquid chromatography and Inductively Coupled Plasma Optical Emission Spectroscopy. Pasture quality, assessed by total digestible nutrients (TDN), peaked in October. Egg protein quality met USDA “Grade AA” standards every month except August (p > 0.001). The highest yolk pigmentation score was recorded in December (9.5 ± 1.3; p < 0.001). Vitamin A levels were significantly greater in late summer (p < 0.001), while vitamin E gradually increased across the season, reaching its highest value in November (118.1 ± 24.0 µg/g fresh yolk; p < 0.001). Carotenoid concentrations were elevated in mid-summer and late autumn (p < 0.001). Total omega-3 fatty acids were significantly higher in September and October than in mid-summer and late fall, while the n-6:n-3 ratio was lowest in early summer, and fall compared to July (p < 0.001). Sparse partial least squares discriminant and random forest analyses demonstrated that eggs produced from September to November contained higher levels of vitamins A and E, greater essential omega-3 fatty acids, and a more favorable n-6:n-3 balance than eggs from other months. These findings highlight the need to account for seasonal variability in pasture-based systems and suggest targeted management practices could enhance year-round nutritional quality, supporting both consumer health and sustainable food production.

## Introduction

Consumer demand is shifting away from conventional production systems, such as cage-free or caged systems, and moving toward more sustainable alternatives—including pasture-based and small-scale backyard operations [1]. In a recent survey of U.S. consumers, 86% of respondents had purchased at least one animal product with welfare associated labels, such as “pasture-raised” [2]. Many consumers are also turning to local sources like farmers’ markets and backyard producers, which often rely on pasture-based methods that emphasize both animal welfare and environmental stewardship.

Pasture-raised egg farming has gained significant attention due to its emphasis on animal welfare, sustainability, and the production of nutrient-dense eggs, distinguishing it from conventional farming systems that rely heavily on confined animal operations and grain-based feed. Moreover, pasture-raised egg production is aligned with regenerative agricultural practices which focus on fostering a symbiotic relationship between chickens, forage, and the environment. These systems emphasize soil health, biodiversity, and nutrient cycling [3]. Hens in these systems have access to a variety of plants and insects, contributing nutrients that are otherwise absent or limited in conventional feed. The nutrient composition of the pasture becomes a critical determinant of egg quality, as hens obtain key fatty acids, antioxidants, and vitamins directly from their surroundings. Numerous studies have highlighted how forage botanical composition, environmental conditions, and hen foraging behavior can influence the nutritional quality of eggs and poultry products, reinforcing the complexity and ecological sensitivity of pasture-based systems [4–7].

Recent literature has made substantial contributions to the understanding of pasture-based poultry systems. A comparative study on conventional, organic, and “organic-plus” systems (increased pasture access) demonstrates that outdoor systems significantly improve animal welfare, particularly by supporting more natural behavioral patterns such as foraging and exploration [8,9]. Further, the organic-plus group produced eggs with improved yolk color, shell weight, and antioxidant content, including higher concentrations of α-tocopherol and carotenoids [9]. Their research also highlights how the nutritional quality of pastures directly affects the transfer of antioxidants and polyunsaturated fatty acids (PUFAs) to poultry products [10]. Chickens raised with access to fresh forage displayed higher omega-3 fatty acid levels, more favorable n-6:n-3 ratios, and improved antioxidant profiles [4,10]. Castellini et al. found that forage intake and nutrient deposition in eggs were highest during spring, when pasture quality peaked; reinforcing the idea of seasonal variations [9]. Further, Castellini et al. has also begun to assess the long-term environmental effects of poultry grazing on soil health. In a 20-year study of free-range chickens in olive groves, they found that moderate grazing maintained or improved soil organic carbon and microbial activity, whereas high stocking densities led to vegetation loss and reduced soil fertility near poultry houses [11]. This underscores the need for rational grazing management to balance productivity with ecological sustainability.

Eggs from pasture-raised systems offer significant nutritional advantages over those produced in conventional systems, such as cage-free or caged systems, where hens are not required to have outdoor access [1,12–15]. Pasture-raised eggs are generally more nutrient-dense compared to eggs from conventional caging systems, with one study reporting twice the vitamin E content and 2.5 times more omega-3 (n-3) fatty acids, contributing to a more favorable omega-6: omega-3 ratio [1]. The increased antioxidant, omega-3 fatty acid, and vitamin content in pasture-raised eggs is beneficial for human health, as consuming these nutrients supports immune function, reduces inflammatory cardiovascular diseases, and mitigates the harmful effects of oxidative stress [16–18]. The high lipid content of egg yolks enhances the absorption of fat-soluble vitamins and antioxidants. Minerals in egg yolk including iron, phosphorus, selenium, and zinc are present in highly absorbable forms that are less affected by inhibitors than those in plant sources, making egg yolks a particularly bioavailable source of these nutrients [19,20]. The inclusion of forages in the hens’ diet contributes higher antioxidant and polyunsaturated fat content, particularly n-3 fatty acids, compared to conventional corn- and soy-based feeds that are usually lower in antioxidants and higher in omega-6 (n-6) fatty acids. In summary, the nutrient profile of eggs is highly responsive to dietary changes [1,21].

Despite the benefits of pasture-based systems, seasonal variation in forage quality and composition poses challenges for consistent egg nutrient profiles. As plants mature, the leaf-to-stem ratio decreases, increasing acid detergent fiber (ADF) and neutral detergent fiber (NDF) while reducing digestible protein—essential for productive laying systems [22]. Lower total digestible nutrients (TDN), a key indicator of pasture quality, can negatively impact hens’ growth, egg production, and nutrient absorption [23]. For example, alfalfa is associated with higher forage phenolic content, and orchard grass is linked to lower total forage carotenoids [21]. Chickens’ preferences influence forage consumption, where grass species, orchard grass and alfalfa have demonstrated highest palatability [24]. Additionally, environmental factors such as rainfall, temperature, and soil conditions further influence forage quality [22,25]. Excess rainfall can leach nutrients from plants, while prolonged high temperatures accelerate the degradation of fatty acids and antioxidants like vitamin E and carotenoids [25]. Thus, understanding how seasonal and environmental variability affect forage quality is essential for optimizing pasture-based systems.

Although the effects of hen diet on egg composition are well documented, there remains a gap in the literature regarding how seasonal shifts in forage quality impact egg nutrients in pasture-based systems. Understanding these dynamics is crucial, as they could inform best practices for improving egg quality year-round in pasture-based systems. Therefore, the objective of this study was to document changes in the nutrient composition of forage and eggs in a Southern Ohio pasture-based system for layer hens across a grazing season. Further, we investigated how changes in egg nutrient composition connect to variations in weather, soil quality, and forage composition, and identified key discriminating factors contributing to seasonal variation in egg nutrients.

## Materials and methods

### Chemicals

A gas chromatography–mass spectrometry (GC-MS) reference standard curve was created using the Supelco 37 Component FAME Mix (Sigma-Aldrich, St. Louis, MO, USA), along with individual standards, including mead acid, docosatetraenoic acid (DTA), n-3 docosapentaenoic acid (DPA), n-6 DPA, and palmitelaidic acid (Cayman Chemical, Ann Arbor, MI, USA). Branch chain fatty acids (BCFAs) were quantified using Mixture BR 3 (Larodan AB, Solna, Sweden), while conjugated linoleic acid (CLA) isomers were quantified using the CLA reference standard UC-59M (Nu-Chek Prep, Elysian, MN, USA). Dichloromethane was obtained from VWR Chemicals (Radnor, PA, USA). All other chemicals were purchased from Sigma-Aldrich (St. Louis, MO, USA), unless otherwise noted. All reagents used were HPLC grade unless otherwise noted, with isooctane being GC grade.

### Diet characteristics and sample collection

This study was conducted across one grazing season (May-December 2022) at a privately managed farm in Southern Ohio (39.22°N, 84.34°W; 269 m elevation), where laying hens were rotated every 4 weeks between three 0.25 acre2 (1011.71 m2) fenced pastures. The farm was independently operated for production and not managed for research. Samples were collected during routine farm operations without experimental intervention or animal monitoring and shipped to Michigan State University for analysis. From May to September, the flock consisted of approximately 300 Comet hens; hens were around one year old at the start of collection. However, the flock size was drastically reduced by September due to predation and a high mortality rate. In response to these losses, Black Sex-linked hens, at an age of 16 weeks, were introduced in October, replacing the Comet hens.

In rotation with grass-fed cattle, hens were rotated every 4 weeks across three fresh pastures. In addition, hens had free access to a standard layer hen feed all season (Table 1). The layer hen feed was sampled three times from a well-mixed bin of feed at the beginning and end of the grazing season each year for a total of n = 6 replicates. Layer hen feed samples were freeze-dried and ground with dry ice to pass a 1 mm screen in a Wiley mill (Arthur H. Thomas, Philadelphia, PA, USA) and stored at −80 °C.

**Table 1:** Composition of the layer hen feed.

Guaranteed Analysis		Nutrition Requirement^1^
Crude Protein (Min)	16.00%	15.00%
Lysine (Min)	0.85%	0.69%
Methionine (Min)	0.35%	0.30%
Crude Fat (Min)	3.50%	ND
Crude Fiber (Max)	9.00%	ND
Calcium (Min)	3.25%	3.25%
Calcium (Max)	3.75%	
Phosphorus (Min)	0.70%	ND
Salt (Min)	0.25%	0.15%
Salt (Max)	0.75%	
Selenium (Min)	0.30 ppm	0.60 ppm
Vitamin A (Min)	882.00 IU/100 g	3,000.00 IU/100 g
Vitamin D3 (Min)	331.00 IU/100 g	300.00 IU/100 g

**Ingredients:** Wheat Midds, Oats, Barley, Organic Non-GMO Soybean Meal, Calcium Carbonate, Fish Meal, Kelp Meal, Salt, Monocalcium Phosphate, Brewers Grain Yeast, Lactobacillus acidophilus, Enterococcus faecium, Aspergillus oryzae, Bacillus subtilis, Bacillus licheniformis, Yucca schidigera, DL-Methionine, Vitamin A Supplement, Vitamin D3 Supplement, Vitamin E Supplement, Menadione Sodium Bisulfite Complex, Niacin, Riboflavin, D-Calcium Pantothenate, Pyridoxine Hydrochloride, Folic Acid, Zinc Amino Acid Chelate, Potassium Amino Acid Complex, Magnesium Amino Acid Chelate, Manganese Amino Acid Chelate, Copper Amino Acid Chelate, Vitamin B12 Supplement, Ferrous Sulfate, Manganese Oxide, Copper Sulfate, Sodium Selenite, Zinc Oxide, Choline Chloride, Ethylenediamine Dihydroiodide, Selenium Yeast.

^1^Represents layer hen intake requirements defined by the Nutrient Requirements of Poultry: Ninth Revised Edition, 1994 [27]. ND, not defined; IU, international unit.

A total of 8 collections of forage, soil, eggs, and weather data were conducted from May to December, at 4-week intervals. Each month, before hens were given access to the pasture, forage height and composition were assessed. Ten hoops (1/2 m2) were randomly tossed across the pasture, and species percent coverage and pre-graze forage height were recorded from the center of each hoop. The same method was used to measure post-graze height after the hens were moved off the pasture, providing an estimate of forage intake across the month.

Then, when the hens were given access to the pasture, forage and soil samples were collected. To collect the forage, nine randomly selected 0.25 m2 quadrats were clipped to a 1 cm stubble and thoroughly mixed. This process was repeated 3 times to create n = 3 replicates of forage per month. Forage samples were promptly placed in a –20 °C freezer until delivery to the laboratory. Then, forage samples were freeze-dried and ground with dry ice to pass a 1 mm screen in a Wiley mill (Arthur H. Thomas, Philadelphia, PA, USA) and stored at −80 °C under nitrogen. At the same time, soil samples were collected. Using a soil probe, 15–20 subsamples were randomly taken in a zig-zag fashion from the pasture area and mixed in a bucket. This process was repeated 3 times to create n = 3 replicates of soil per month.

After the hens had access to the pasture for several days, 36 eggs were randomly collected. Upon arrival at the laboratory, n = 24 eggs were randomly chosen for analysis. Finally, weather data, including daily, monthly, and 30-year normal average temperature and total precipitation, was obtained from the U.S. Department of Commerce National Centers for Environmental Information [26].

### Soil analysis

Soil samples were analyzed under the organic matter and general soil profile packages at a commercial laboratory provided through Michigan State University (East Lansing, MI, USA). Soil pH was assessed using a standard pH meter. Additionally, organic matter and ash content were determined using the loss on ignition (LOI) method using a muffle furnace. Mineral content was assessed after the LOI ash product for Inductively Coupled Plasma Optical Emission Spectroscopy (ICP-OES) quantification.

### Forage and layer hen feed proximate analysis

Forage and layer hen feed proximate analysis was conducted at the DairyOne Forage Laboratory in Ithaca, NY, USA. Forage and feed moisture content was assessed using a forced air oven adapted from AOAC 991.01 and AOAC 930.15 methods, respectively [26]. Crude protein (CP), ADF, lignin, crude fat, and ash content were assessed using AOAC methods 990.03, 973.18, 973.18, and 954.02, respectively [28]. Forage and feed NDF content was assessed based on methods adapted from Van Soest et al [29]. For the starch analysis, forage and feed samples were enzymatically digested into glucose using glucoamylase, then the resulting glucose was quantified indirectly using hydrogen peroxide equivalents measured by the YSI 2700 Select Biochemistry Analyzer. Metabolizable energy, digestible energy (DE), and TDN were calculated using equations as previously described [30].

### Egg physical characteristics

Egg physical characteristics were measuredas previously reported [12,13]. Egg, yolk, and shell weight were recorded, and albumen weight was determined by subtraction. Albumen height was determined using a micrometer. Haugh units were determined from the recorded egg weight and albumen height (Haugh unit = 100 x log (albumen height (mm) + 7.57–1.7 x egg weight 0.37) [31]. A colorimeter was used to quantify yolk color using the L*a*b (L* scale quantifies whiteness, a*, redness, and b*,yellowness) [32]. Yolk color was also rated from 1 to 14 using the DSM yolk color fan (DSM Nutritional Products, Basel, Switzerland) (1 for pale yellow-16 for deep orange). Lastly, yolks were freeze-dried, powdered, and kept under nitrogen at −80 °C. Every two egg yolks were thoroughly mixed, creating n = 12 replicates per month for subsequent analyses.

### Fatty acid analysis

Briefly, a modified version of the microwave-assisted extraction method by Bronkema et al [33] was used to extract fatty acids using 400 mg of egg yolk, forage, or layer hen feed samples and 8 mL of a 4:1 (v/v) ethyl acetate:methanol solution with 0.1% butylatedhydroxy toluene (BHT) as an antioxidant. Fatty acids were extracted in a CEM Mars 6 microwave (CEM Corp., Matthews, NC, USA) using the following microwave parameters: 55 °C for 15 min with an initial ramp of 2 min at 400 W maximum power. Samples were then filtered and prepared as previously described to obtain the extracted oil [12,13].

Methylation described by Sergin et al [12] modified from Jenkins [34] was conducted for the creation of fatty acid methyl esters (FAMEs). Two mg of extracted oil were combined with 500 µL toluene and 20 µg of methyl-12-tridecenoate (U-35M, Nu-Chek Prep, Elysian, MN, USA) as an internal standard. Base-catalyzed methylation was conducted using 2 mL of anhydrous potassium methoxide (0.5 N) at 50 °C for 10 min. Then, acid-catalyzed methylation was conducted using 3 mL of methanolic HCl (5%) at 80 °C for 10 min. Two mL of HPLC water were added, then FAMEs were extracted twice using 2 mL of hexane. Extracted FAMEs were resuspended in 1 mL of isooctane and stored at −20 °C until GC-MS analysis.

FAMEs were separated on an HP-88 column (100 m, Agilent Technologies, Santa Clara, CA) using a Perkin Elmer 680/600 GC-MS (Waltham, MA, USA). Temperature parameters previously described by Kramer et al. [35] to improve fatty acid isomer separation. Injections (1 µL) included a 30:1 split and a splitless run (0.75 min hold). MS settings: 70 eV electron energy, 180 °C transfer line and ion source, full scan (m/z 70–400).

FAMEs were identified using MassLynx (4.1 SCN 714; Waters Corp., Milford, MA, USA) by comparison to reference standards [35]. Quantification was based on extracted ion chromatograms and standard curves. Results were expressed as percent total fatty acids and g/100 g yolk.

### Phenolic analysis

Phenolic compounds were extracted from lyophilized yolk, forage, and feed samples using previously published methods involving sequential methanol- and acetone-based solvent extractions [12,13]. Briefly, 100μL Folin-Ciocalteu reagent and 800 μL 5% sodium bicarbonate were added to a gallic acid standard curve (1 mg/mL to 0.002 mg/mL) and to a 100 μL portion of the supernatant. Samples were heated at 40 °C for 30 min, cooled at RT for 10 min, and were plated in triplicate in a 96-well plate. Samples were scanned using a microplate reader (Bio-Tek, Winooski, VT, USA) at 765 nm, compared against the standard curve, and reported as mg of gallic acid equivalents (GAE) per g of fresh egg yolk, forage, or feed.

### Carotenoid analysis

For egg yolks, 0.5 g of lyophilized egg yolk sample was combined with 5 mL of cold acetone (0.05% BHT) and homogenized. Samples were vortexed for 2 min, then ultrasonicated in a water bath for 5 min, and centrifuged for 15 min (1200 g, 4 °C). The supernatant was analyzed using a UV-Vis Double Beam Spectrophotometer (VWR, Radnor, PA, USA) at 450 nm against an acetone blank. Total carotenoid content was calculated according to Biehler et al [36]. using an ε of 140663 L/mol for beta-carotene in acetone and was expressed as µg of beta-carotene per g of fresh egg yolk.

For the forage and layer hen feed, 2 g of ground sample were combined with 20 mL of 70% aqueous acetone. The tubes were shaken for 30 min and centrifuged for 20 min at 840 g and 4 °C. The supernatant was recovered in a new tube. The extraction was repeated with an additional 20 mL of 70% aqueous acetone and the supernatants were pooled. Using the spectrophotometer, carotenoid and chlorophyll content of the supernatants were assessed in glass cuvettes at three wavelengths (663, 646, and 470 nm). Chlorophyll A, chlorophyll B, and total carotenoids were calculated using equations as previously described [37].

### Vitamin A and E analysis

The vitamin content of egg yolk, forage, and layer hen feed samples was assessed using the Veterinary Diagnostic Laboratory at Michigan State University (East Lansing, MI) using AOAC official method 2001.13 [28].

### Mineral analysis

For egg yolks, 0.10 g of powdered yolk was predigested in borosilicate glass tubes with 3 mL of a concentrated ultrapure nitric and perchloric acid mixture (60:40 v/v) for 16 hr at RT. Samples were then heated incrementally in a digestion block to 120 °C for 4 h, followed by 2 h at 120 °C with an additional 2 mL of nitric acid. The temperature was then increased to 145 °C for 2 h and finally to 190 °C to evaporate remaining liquid. Digested samples were resuspended in 10 mL ultrapure water and analyzed using Inductively coupled plasma-atomic emission spectrometry (ICP-AES) (Thermo iCAP 6500 Series) with quality control standards for every 10 samples. Yttrium (0.50 μg/mL, final concentration) was added as an internal standard to ensure accuracy and correct for matrix interference.

For the forage and layer hen feed, 0.5 g of forage and layer hen feed samples were digested in 10 mL of a 4:1 (v/v) nitric:hydrochloric acid solution, followed by an additional 10-min digestion with 1 mL of 30% hydrogen peroxide. Digestions were performed using a CEM Mars 6 microwave system (CEM Corp., Matthews, NC, USA) under the following parameters: a 10-min ramp to 135 °C held for 3 min at 1500 W, followed by a 12-min ramp to 200 °C held for 15 min at 1600 W. Post-digestion, vessels were diluted to 50 mL, and aliquots were analyzed for mineral content using ICP-OES with a Thermo iCAP Pro XP radial spectrometer. For water analysis, 35 µL of concentrated nitric acid was added to 14 mL of water, mixed, and aspirated for ICP-OES measurement.

### Egg yolk cholesterol analysis

Briefly, cholesterol was extracted from 0.5 g of freeze-dried powdered egg yolk by dilution using 9 mL of 2% (w/v) NaCl. Each replicate was vortexed for two min and shaken at 37 °C for 2 hr. After solubilization, 0.5 mL of the solution was further diluted in 9.5 mL of 2% (w/v) and vortexed for 1 min. Then, the extraction solution was filtered through a 0.45 µm syringe filter to isolate cholesterol. 50 µl of the filtered, diluted, solution was calculated to contain 3−6 µg of cholesterol. Quantification of the extracted cholesterol was determined colorimetrically following instructions using Cholesterol Quantification Assay kit (catalog: CS0005−1KT) produced by Sigma-Aldrich (Burlington, MA).

### Statistical analysis

Means and standard deviations for each characteristic were calculated by month. To assess if egg yolk and forage nutrient content differed by month across the season, a one-way analysis of variance (ANOVA) and Tukey’s Honestly Significant Difference (HSD) test for significance was carried out using RStudio (R Core Team, Vienna, Austria). Results were considered significant at p < 0.05. Values under the limit of detection (LOD) were treated as zeroes. Additionally, a Spearman correlation analysis was carried out to explore how different factors were connected using the RStudio packages: ggplot2, reshape2, Hmisc, RColorBrewer, corrplot, showtext, readxl.

Further, MetaboAnalyst 5.0 (metaboanalyst.ca) was used to carry out sparse partial least squares discriminant analysis (sPLS-DA) to visualize monthly groupings. Random forest (RF) analysis was used to identify which nutrients were the strongest predictors for the separation of each month using 500 trees using OOB values with randomness [38]. Both analyses were conducted using yolk and forage antioxidants (total phenolics, total carotenoids, beta-carotene, vitamin A, and vitamin E) and fatty acids (% of total), and yolk cholesterol, with no data transformation or normalization necessary. Yolk mineral content was excluded from the sPLS-DA and RF analyses, as the minerals contributed minimally to the daily recommended intake for essential minerals, making them insignificant for this analysis.

## Results

### Weather

**Fig 1:**
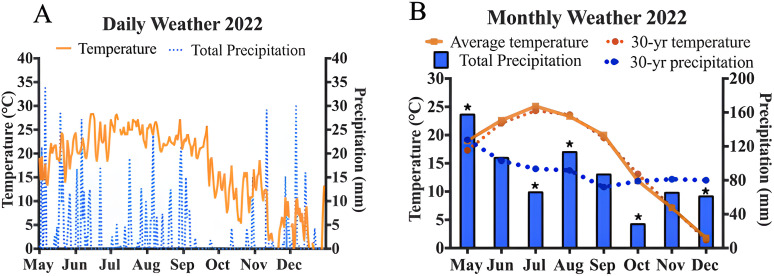
Weather trends across the 2022 grazing season. **(A)** Daily average temperatures and total precipitation **(B)** Monthly average temperature and total precipitation and their comparison to the 30-year normal. *Signifies average monthly total precipitation that is greater than three standard deviations from the 30-year normal.

The daily and monthly average temperature and total precipitation are shown in Fig 1. From May to December 2022 in Southern Ohio, daily temperatures followed expected seasonal patterns, increasing after May, peaking in July and August, and gradually decreasing as the season progressed. The monthly average temperature was closely aligned with the 30-year normal. The highest total precipitation was recorded in May and September. The monthly average precipitation differed from the 30-year normal throughout the season. The months of May, August, and September experienced higher total precipitation compared to the normal, while July and October had notably lower amounts.

### Soil composition

Changes in the soil composition are shown in Table 2. Across the laying season, the soil pH and mineral content were sufficient to maintain forage quality [39]. While several characteristics remained relatively stable across the season, such as pH, lime index, and organic matter, the mineral content fluctuated by month.

**Table 2:** Characteristics of the soil by month^1^.

Parameter	May	Jun	Jul	Aug	Sep	Oct	Nov	Dec	*p*-value^2^
pH	6.40 ± 0.01	6.70 ± 0.26	6.77 ± 0.32	6.43 ± 0.32	6.57 ± 0.15	6.73 ± 0.21	6.53 ± 0.15	6.23 ± 0.06	0.088
Lime index	70.00 ± 0.01 c	70.00 ± 0.01 c	70.00 ± 0.01 c	69.00 ± 0.01 d	71.00 ± 0.01 b	71.00 ± 0.01 b	72.33 ± 0.58 a	69.00 ± 0.01 d	<0.001
Phosphorus (ppm)	18.00 ± 4.36 bc	13.67 ± 3.51 cd	14.67 ± 1.53 cd	5.00 ± 1.73 e	7.00 ± 1.00 de	13.33 ± 3.21 cd	47.33 ± 3.51 a	24.33 ± 1.15 b	<0.001
Potassium (ppm)	164.00 ± 30.51 b	71.67 ± 18.50 b	210.67 ± 51.19 ab	100.33 ± 26.16 b	97.00 ± 16.46 b	197.33 ± 12.66 ab	326.33 ± 121.71 a	157.33 ± 34.67 b	<0.001
Magnesium (ppm)	228.33 ± 16.20 ab	160.67 ± 7.77 c	231.67 ± 13.58 a	192.67 ± 13.05 abc	195.33 ± 14.50 abc	237.67 ± 35.57 a	221.67 ± 6.35 ab	181.67 ± 15.37 bc	<0.001
Calcium (ppm)	1413.33 ± 73.33 ab	1591.33 ± 102.05 ab	1706.67 ± 154.78 a	1564.33 ± 249.5 ab	1387.33 ± 93.11 ab	1680.33 ± 178.21 a	1552.00 ± 27.87 ab	1237.67 ± 21.55 b	0.008
CEC (meq/100 g)	9.40 ± 0.44 ab	9.50 ± 0.52 ab	11.00 ± 0.78 a	10.50 ± 0.72 ab	8.83 ± 0.55 b	10.90 ± 1.23 a	10.43 ± 0.42 ab	9.33 ± 0.25 ab	0.007
% of Exchangeable bases	7.08 ± 1.59 d	7.96 ± 1.20 bcd	8.62 ± 1.41 abc	9.00 ± 1.38 ab	7.33 ± 1.88 cd	8.38 ± 2.79 abcd	8.79 ± 0.88 abc	9.54 ± 1.38 a	<0.001
% Potassium	4.50 ± 0.98 ab	1.97 ± 0.55 b	5.00 ± 1.59 ab	2.60 ± 0.30 ab	2.87 ± 0.64 b	4.67 ± 0.25 ab	7.93 ± 2.63 a	4.97 ± 0.93 ab	0.001
% Magnesium	20.27 ± 0.50 a	14.13 ± 0.58 c	17.57 ± 0.21 ab	16.73 ± 1.95 bc	18.43 ± 0.81 ab	18.13 ± 1.07 ab	17.70 ± 0.98 ab	18.33 ± 0.90 ab	<0.001
% Calcium	75.23 ± 0.55 cd	83.90 ± 1.13 a	77.47 ± 1.72 bcd	80.63 ± 1.67 ab	78.67 ± 0.99 bc	77.20 ± 0.95 bcd	74.33 ± 1.76 d	76.40 ± 1.82 cd	<0.001
Organic matter (%)	4.47 ± 0.21 ab	4.23 ± 0.35 ab	4.23 ± 0.06 ab	4.70 ± 0.20 a	4.47 ± 0.06 ab	4.67 ± 0.12 a	4.13 ± 0.15 b	4.03 ± 0.15 b	0.003

^1^Means ± standard deviation (****n**** = 24 eggs per month).

^2^Results of one-way ANOVA. a-e, Means within a row with different letters significantly differ (p < 0.05). ppm, parts per million.

CEC, Cation exchange capacity.

Additionally, several trends were observed when comparing soil characteristics with forage mineral content (S1 Table). For example, phosphorus levels in the soil generally decreased over the season, dropping from 18.00 ppm in May to 5.00 ppm in August, before rising again in November (p < 0.001). The forage phosphorus levels followed a similar pattern, decreasing from 0.04% in May to 0.03% by November (p = 0.035).

### Forage composition and height

**Fig 2:**
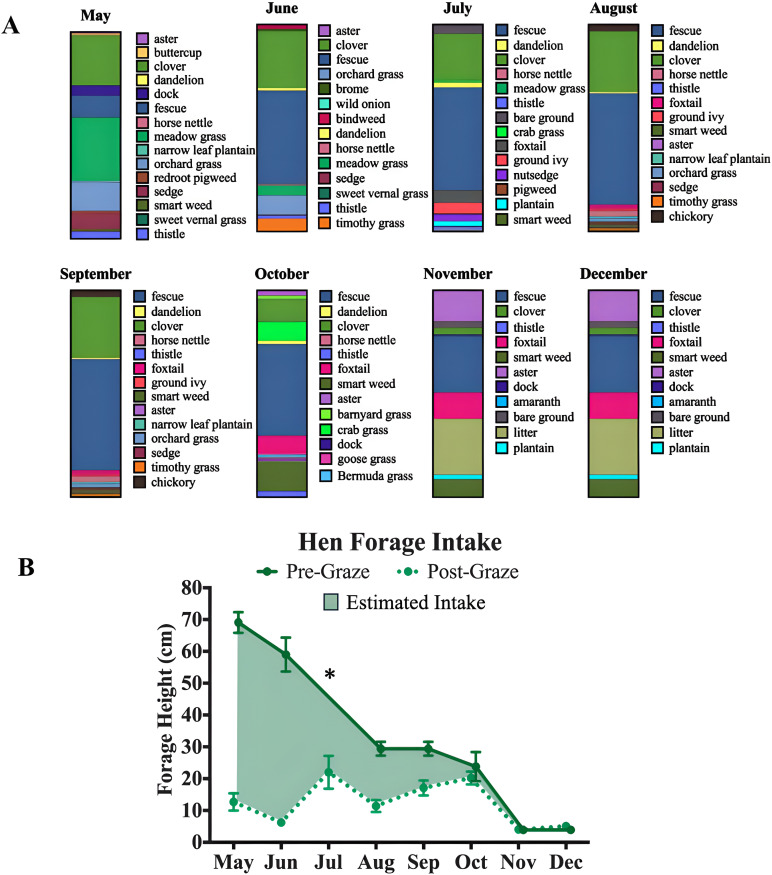
Monthly forage composition and estimated hen forage intake. (A) Illustrates the proportion and types of species that make up the monthly forage composition. **(B)** Height of forage before and after the hens grazed representing intake estimates across the year. *Pre-Graze height data not available.

The forage composition varied greatly across the laying season (Fig 2). The pasture featured a diverse mix of species, with the most prevalent being clover (Trifolium repens), fescue (Festuca), thistle (Cirsium), smartweed (Persicaria lapathifolia), and aster (Tripolium pannonicum). Additionally, the months with highest seasonal temperatures, July, and August, had the most plant diversity despite the impact of seasonal changes on the forage height. The difference between pre- and post-graze heights varied throughout the year. During peak summer, post-graze heights were especially low (e.g., June’s drop from 59.1 cm to 6.2 cm). From September to December, forage consumption also shifted, leading to smaller differences between pre- and post-graze heights and a reduced variety of forage as the season was ending.

### Forage and layer hen feed nutrient composition

**Fig 3:**
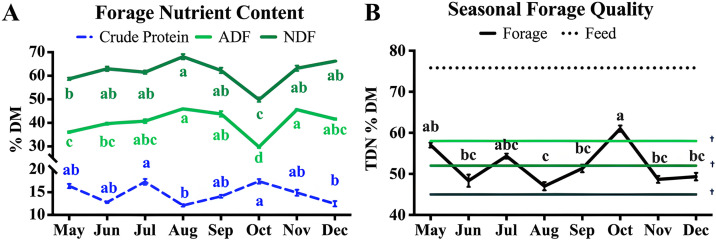
Seasonal changes in the forage quality and proximate analysis. Means and standard error of the mean (SEM) are shown. **(A)** Forage proximate analysis data **(B)** Forage quality based on total digestible nutrients. ADF; acid detergent fiber, NDF; neutral detergent fiber, TDN; total digestible nutrients. Results of one-way ANOVA. a-e, means within a row with different letters significantly differ **p* *< 0.05. † Indicates Low (45%), Medium (52%), and High (58%) quality forage based on TDN (% DM).

Proximate analysis values for monthly forage and feed are displayed in Fig 3 and S1 Table. In this study, the highest crude protein (CP) levels were observed in July (17.30% DM) and October (17.40%), with lower levels in August (12.10%) and December (12.47%) (p = 0.003). Throughout the grazing season, ADF values ranged from 36% to 45% DM, with the lowest values in the early season and the highest in August, indicating increasing plant maturity by the end of summer (p < 0.001). Within this pasture raising system, TDN ranged widely across the grazing season from 47 to 61% DM (p < 0.018). Low quality forage TDN values fall within 45–52% DM, while mid quality forage ranges 52–58% DM, and high-quality forage exceeds 58% [23]. Overall, the feed had a higher availability of digestible nutrients compared to the forage.

Additionally, forage and feed fatty acid profiles are presented in S2 and S3 Tables. Total forage fatty acids ranged from 3.625 g per 100 g in December to 14.801 g per 100 g in July (p = 0.121), whereas feed samples contained significantly more fat, averaging 164.131 g per 100 g of sample. Forage alpha-linolenic acid (ALA) content peaked in July at 6.681 g per 100 g and gradually decreased as the season progressed, reaching a low of 0.761 g per 100 g in December (p = 0.761). The feed samples contained higher total n-3 fatty acid levels (13.422 g per 100 g), nearly double the highest forage n-3 content (6.711 g per 100 g). Feed n-3 fatty acids primarily comprised docosahexaenoic acid (DHA) and DPA n-3. Forage and feed antioxidant data are detailed in S4 Table. Forage carotenoid content was highest in May (765.92 µg per g) and lowest in November (73.06 µg per g) (p = 0.001) but remained significantly higher than feed carotenoid levels, which averaged 14.47 µg per g. Total forage phenolic content was generally higher than feed phenolic content, except in November.

### Egg characteristics

Significant differences in the egg characteristics are shown in Table 3. Across the grazing season, significant differences were observed in egg weight ranging from 53 to 60 g (p = 0.004). Eggs from July and November were significantly larger compared to September (p = 0.004). The yolk fan values ranged from 7.08 in May to 9.54 in December (p < 0.001), with the highest value observed in the peak summer months and December. Based on colorimeter values, yolk colors were significantly lighter in June and September, had a more prominent yellow color in the month of October, and had strongest red influence in the month of August. Haugh units significantly varied, ranging from 61.20 in August to 88.28 in October (p < 0.001).

**Table 3:** Physical characteristics of the eggs by month^1^.

Parameter	May	Jun	Jul	Aug	Sep	Oct	Nov	Dec	*p*-value^2^
Egg weight (g)	56.57 ± 5.43 ab	58.16 ± 4.97 ab	60.70 ± 6.73 a	58.85 ± 9.93 ab	53.39 ± 6.69 b	57.73 ± 6.70 ab	60.38 ± 4.94 a	57.31 ± 4.24 ab	0.004
Shell weight (g)	5.53 ± 0.54 abc	5.73 ± 0.48 abc	5.75 ± 0.84 abc	5.91 ± 1.05 ab	5.28 ± 0.79 c	5.45 ± 0.82 bc	5.92 ± 0.41 ab	6.12 ± 0.49 a	0.001
Yolk weight (g)	12.80 ± 1.00 cd	13.00 ± 0.90 cd	14.38 ± 2.14 ab	14.02 ± 1.84 abc	12.02 ± 1.88 d	13.15 ± 1.65 bcd	13.88 ± 1.22 abc	14.73 ± 1.09 a	<0.001
Dried yolk weight (g)	6.58 ± 0.56 bc	6.68 ± 0.51 abc	7.32 ± 1.13 a	6.92 ± 0.91 ab	6.10 ± 0.99 c	6.71 ± 0.93 abc	7.11 ± 0.69 ab	7.31 ± 0.61 a	<0.001
Albumin weight (g)	38.25 ± 4.79 ab	39.42 ± 4.15 ab	40.58 ± 5.08 a	38.92 ± 7.66 ab	36.10 ± 4.71 b	39.13 ± 4.92 ab	40.57 ± 3.99 a	36.46 ± 3.26 ab	0.010
Albumin height (μm)	7.28 ± 0.95 ab	6.61 ± 1.04 bc	5.85 ± 1.39 cd	4.45 ± 1.21 e	7.04 ± 1.44 ab	7.73 ± 1.13 a	6.63 ± 1.09 bc	5.55 ± 0.99 d	<0.001
Haugh unit	86.21 ± 4.63 a	81.27 ± 6.60 ab	74.04 ± 12.01 b	61.20 ± 18.34 c	85.07 ± 9.43 a	88.28 ± 6.51 a	80.56 ± 7.65 ab	73.81 ± 7.35 b	<0.001
Yolk color fan^3^	7.08 ± 1.59 d	7.96 ± 1.20 bcd	8.62 ± 1.41 abc	9.00 ± 1.38 ab	7.33 ± 1.88 cd	8.38 ± 2.79 abcd	8.79 ± 0.88 abc	9.54 ± 1.38 a	<0.001
Colorimeter^4^ (L)	67.55 ± 3.04 ab	68.90 ± 1.86 a	68.06 ± 2.52 ab	66.71 ± 2.55 ab	68.86 ± 2.98 a	66.07 ± 3.94 b	67.68 ± 1.21 ab	65.78 ± 2.35 b	<0.001
Colorimeter (a)	10.68 ± 3.27 d	14.83 ± 2.80 bc	15.86 ± 3.29 abc	19.26 ± 3.29 a	14.58 ± 5.02 c	17.74 ± 6.33 abc	18.20 ± 1.56 ab	17.34 ± 3.33 abc	<0.001
Colorimeter (b)	56.53 ± 4.02 d	61.56 ± 2.94 bc	60.64 ± 2.62 c	64.60 ± 3.62 b	60.57 ± 3.78 c	69.83 ± 4.75 a	61.46 ± 3.69 bc	59.52 ± 4.10 cd	<0.001

^1^Means ± standard deviation (****n**** = 24 eggs per month).

^2^Results of one-way ANOVA.

^3^Yolk color fan was measured on a scale of 1–16 from light yellow to dark orange. a-e, Means within a row with different letters significantly differ (****p**** < 0.05).

^4^Colorimeter numerically assess color gradient (L* scale quantifies whiteness, a*, redness, and b*, yellowness.

### Egg yolk antioxidants

**Fig 4:**
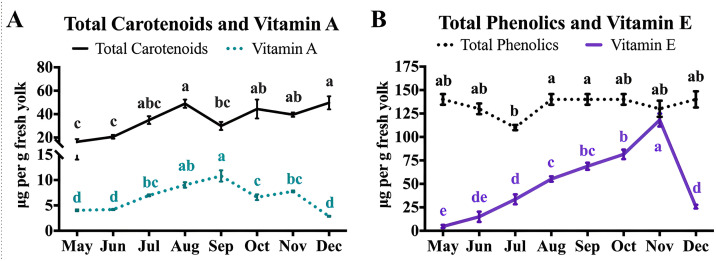
Significant changes in the yolk antioxidant profile. Monthly means and SEM are shown. (A) Changes in the vitamin A and total carotenoid content **(B)** Changes in yolk vitamin E content. Results of one-way ANOVA. a-e, means within a row with different letters significantly differ (p < 0.05).

Changes in the yolk antioxidant profile can be observed in Fig 4 and S5 Table. Significant changes in the yolk antioxidant profile were observed across the season based on vitamin total carotenoids, beta-carotene, and vitamin E content. Total yolk phenolic content remained stable across the year, showing no apparent seasonal variations. (p = 0.019). Vitamin A levels in egg yolks gradually increased throughout the summer, peaking in September (p < 0.001), while vitamin E rose significantly from May to November before dropping sharply in December (p < 0.001). Conversely, total carotenoid levels rose from May, peaking in August, and remained relatively high through October before stabilizing in December (p < 0.001), with similar trends observed with beta-carotene.

### Egg yolk fatty acid profiles

**Fig 5:**
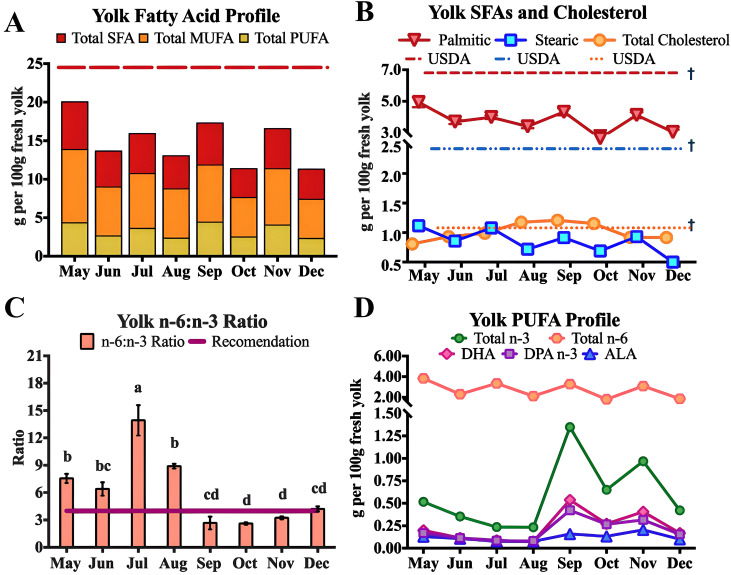
Notable seasonal variations in the yolk FA profile. Monthly means and SEM are shown. (A) Total SFA, MUFA, and PUFA values across the grazing season compared to the USDA expected value for total FA. **(B)** Palmitic, stearic, and total cholesterol across the season compared to expected USDA nutrient content. **(C)** Monthly changes in the n-6: n-3 ratio compared recommendation [17]. **(D)** Seasonal variations in the total and individual omega-3 fatty acid content. SFA, saturated fatty acids; MUFA, monounsaturated fatty acids, PUFA, polyunsaturated fatty acids; † USDA Cage-Free egg yolk expected nutrient value (25.45 g total fat, 6.86 g palmitic acid, 0.104 g stearic acid, and 1.08 g per 100 g) [40]. Results for the yolk SFAs, cholesterol, and the n-6:n-3 ratio as shown as mean ± SEM.

Seasonal variations in yolk fatty acids are presented in Fig 5, Table 4 and S6 and S7 Tables. Significant monthly changes in fatty acid profiles were observed throughout the grazing season, with total fatty acids peaking in May at 20.84 g per 100 g and progressively declining to a low of 11.38 g per 100 g in October, before continuing to decrease through December. These values remained consistently below the expected 28.8 g per 100 g of fresh yolk (p < 0.001). Saturated fatty acids were significantly lower than expected USDA values, with total palmitic acid peaking at 4.98 g per 100 g (p < 0.001) compared to the expected 6.8 g per 100 g, while total stearic acid content consistently fell below the USDA expectation of 2.42 g per 100 g of yolk (p < 0.001) [40]. Across the season, cholesterol ranged from 0.809 g in May to 1.209 g per 100g in September (p < 0.001) peaking halfway through the season. Although slight variations were observed in cholesterol content, overall, the content was close to the expected USDA value of 1.08 g per 100 g of egg yolk [40].

**Table 4:** Egg yolk branched chain and conjugated linoleic fatty acids by month (g of fatty acid per 100 g of fresh egg yolk)^1^.

Fatty Acid	Carbon Number	May	Jun	Jul	Aug	Sept	Oct	Nov	Dec	*p*-value^2^
CLA	9c, 11t 18:2	0.016 ± 0.002 b	0.013 ± 0.002 bcd	0.012 ± 0.002 cd	0.011 ± 0.001 d	0.022 ± 0.006 a	0.017 ± 0.003 b	0.022 ± 0.002 a	0.015 ± 0.002 bc	<0.001
	11t, 13c 18:2	0.009 ± 0.001 c	0.009 ± 0.001 bc	0.009 ± 0.001 bc	0.009 ± 0.001 c	0.012 ± 0.002 a	0.010 ± 0.001 bc	0.011 ± .001 b	0.010 ± 0.001 bc	<0.001
	11t, 13t 18:2	0.032 ± 0.002 bc	0.028 ± 0.003 cde	0.026 ± 0.004 de	0.024 ± 0.002 e	0.046 ± 0.007 a	0.033 ± 0.005 b	0.041 ± 0.003 a	0.031 ± 0.004 bcd	<0.001
	t, t 18:2	0.009 ± 0.001 b	0.010 ± 0.001 b	0.010 ± 0.001 b	0.009 ± 0.001 b	0.011 ± 0.002 a	0.009 ± 0.001 b	0.010 ± 0.001 b	0.009 ± 0.001 b	<0.001
C14:0-*iso*	14:0	LOD	LOD	LOD	LOD	LOD	LOD	LOD	LOD	ND
C15:0-*iso*	15:0	6.812 1.209 a	5.533 ± 0.701 b	5.548 ± 0.539 b	5.789 ± 1.036 ab	6.591 ± 1.447 ab	4.206 ± 1.158 c	6.238 ± 0.677 ab	4.194 ± 0.645 c	<0.001
C15:0-*anteiso*	15:0	9.667 ± 2.024 a	6.939 ± 0.784 b	7.382 ± 0.818 b	6.803 ± 0.819 b	7.26 ± 1.039 b	5.139 ± 1.593 c	7.311 ± 0.783 b	5.307 ± 0.881 c	<0.001
C16:0-*iso*	16:0	3.684 ± 0.957 a	2.576 ± 0.617 bcd	3.271 ± 0.983 ab	2.153 ± 0.303 cde	2.925 ± 0.649 abc	1.675 ± 0.661 e	2.907 ± 0.495 abc	1.76 ± 0.318 de	<0.001
C17:0-*iso*	17:0	3.290 ± 0.899 a	2.288 ± 0.572 bcd	2.996 ± 0.975 ab	1.895 ± 0.274 cd	2.629 ± 0.588 abc	1.457 ±0.560 e	2.565 ± 0.450 abc	1.588 ± 0.283 de	<0.001
C17:0-*anteiso*	17:0	0.317 ± 0.072 a	0.238 ± 0.056 abc	0.215 ± 0.053 bcd	0.211 ± 0.054 bcd	0.243 ± 0.067 abc	0.184 ± 0.101 cd	0.292 ± 0.059 ab	0.147 ± 0.042 d	<0.001
C18:0-*iso*	18:0	10.596 ± 2.424 b	9.742 ± 2.231 b	14.575 ± 5.416 a	9.550 ± 2.941 b	11.161 ± 2.014 ab	8.680 ± 2.516 b	8.934 ± 1.500 b	11.291 ± 2.691 ab	0.006
C18:0-*anteiso*	18:0	0.083 ± 0.022 a	0.057 ± 0.013 bc	0.063 ± 0.015 bc	0.048 ± 0.011 cd	0.067 ± 0.014 ab	0.034 ± 0.015 d	0.058 ± 0.017 bc	0.034 ± 0.006 d	<0.001
Total CLA		0.066 ± 0.005 cd	0.060 ± 0.006 cd	0.057 ± 0.007 cd	0.052 ± 0.004 d	0.083 ± 0.027 a	0.069 ± 0.010 bc	0.083 ± 0.006 ab	0.065 ± 0.008 cd	<0.001
Total BCFA		0.071 ± 0.005 b	0.078 ± 0.010 ab	0.071 ± 0.007 b	0.069 ± 0.006 b	0.084 ± 0.014 a	0.072 ± 0.008 b	0.075 ± 0.004 ab	0.072 ± 0.007 b	<0.001
Total *iso*BCFA		0.055 ± 0.004 b	0.061 ± 0.008 ab	0.055 ± 0.006 b	0.053 ± 0.004 b	0.065 ± 0.013 a	0.056 ± 0.006 ab	0.058 ± 0.003 ab	0.057 ± 0.006 ab	0.001
Total *anteiso*BCFA		0.016 ± 0.001 bc	0.018 ± 0.002 ab	0.016 ± 0.002 bc	0.016 ± 0.002 bc	0.019 ± 0.002 a	0.015 ± 0.002 c	0.016 ± 0.001 bc	0.015 ± 0.002 bc	<0.001

^1^Means ± standard deviation (n = 24 eggs pooled into n = 12 replicates per month).

^2^Results of one-way ANOVA. a-e, Means within a row with different letters significantly differ (****p**** < 0.05).

BCFA, branched-chain fatty acids; CLA, conjugated linoleic acid; FA, fatty acids.

Omega-3 content varied widely throughout the grazing season, with lower levels (0.234 g to 0.516 g per 100g) observed during the late spring and summer months, followed by a significant increase to 1.349 g per 100 g in September (p < 0.001). The n-6:n-3 ratio was closest to the recommended 4:1 during the fall months (p < 0.001). As shown in S3 Table, relative n-6 content exhibited only minor fluctuations across the season, indicating that the lower ratio observed in the fall was primarily driven by the substantial increase in n-3 fatty acids rather than changes in n-6 levels. Changes across the grazing season were observed in the branched-chain (BCFA) and conjugated linoleic acid (CLA) acids in Table 3 and S7 Table. Branch chain fatty acids that were quantified in this pasture-raised system were C15:0-iso, C15:0-anteiso, C16:0-iso, C17:0-iso, C17:0-anteiso, C18:0-iso, and C18:0-anteiso. Total BCFA levels were significantly higher in September compared to May, July, August, October, and December, indicating a seasonal effect on BCFA production (p = 0.002). In this system, CLA was present in the egg yolks in four isomers: cis-9, trans-11, trans-10, cis-13, and trans-trans CLA. Total CLA ranged from 0.21% in May to 0.41% in October (p < 0.001) [41].

### Yolk mineral profile

The yolk mineral profile is reported in S8 Table. Seasonal changes in the yolk mineral profile were observed, with phosphorus, magnesium, and manganese generally peaking during the summer months, particularly in July (p < 0.001 for all). In contrast, sodium levels were notably higher in November and December compared to the rest of the grazing season (p < 0.001). Essential minerals, including calcium, potassium, magnesium, iron, zinc, and selenium, were insufficient throughout the season for the average yolk to be classified as a high source of nutrients [42].

### Correlations between yolk and forage nutrients and seasonal impacts

**Fig 6:**
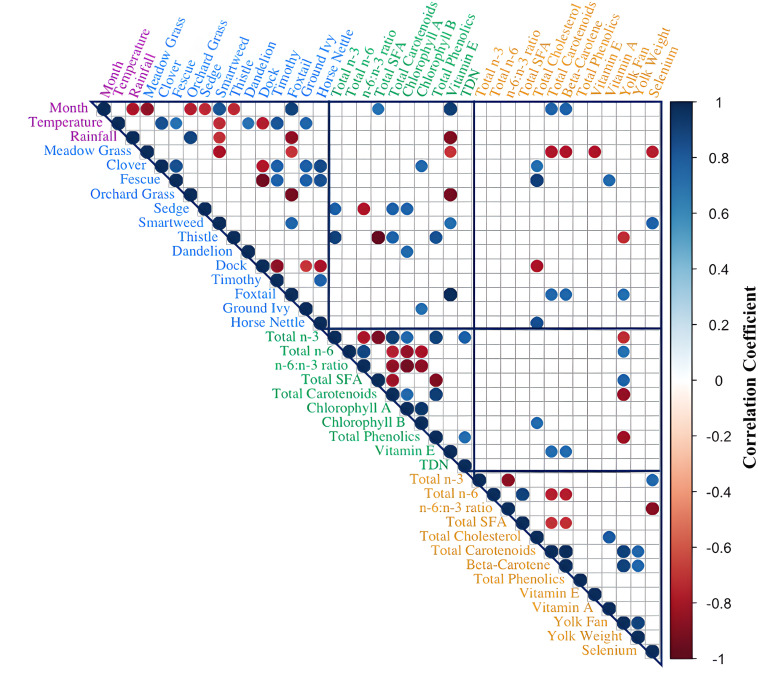
Spearman correlation matrix illustrating significant relationships across monthly averages of egg nutrients, forage nutrients, environmental changes, and forage species parameters (p < 0.05). The color intensity represents the strength of the correlation depicted: Blue represents R coefficient values between 0 to 1, while red represents values between 0 to –1. Text colors distinguish between sample type: purple for environment, blue for forage species present in the pasture, green is assigned to forage nutrients, and yellow to egg nutrients. total omega-3 fatty acids, total n-6; total omega-6 fatty acids, total SFA; total saturated fatty acids; TDN, total digestible nutrients.

In Fig 6, Spearman correlations were carried out across yolk nutrients, forage nutrients, individual forage species, and environmental changes to demonstrate significant relationships across the whole biosystem (p < 0.05). Yolk cholesterol content observed strong positive relationships with orchard grass, fescue, and horse nettle forage species. Yolk total carotenoid and beta-carotene content were significantly associated with the month, forage vitamin E, meadow grass, and foxtail species. Rainfall displays a strong negative relationship with forage vitamin E. The yolk fan score was primarily linked to forage nutrient parameters but unexpectedly showed an inverse relationship with forage phenolics and total carotenoid content. Additionally, among the yolk nutrients most influenced by forage intake, no significant relationships were observed between their levels in the forage (n-3 PUFAs, carotenoids, vitamin E, phenolics) and their corresponding levels in the eggs.

### Yolk, forage, and feed discriminant and random forest analysis

**Fig 7:**
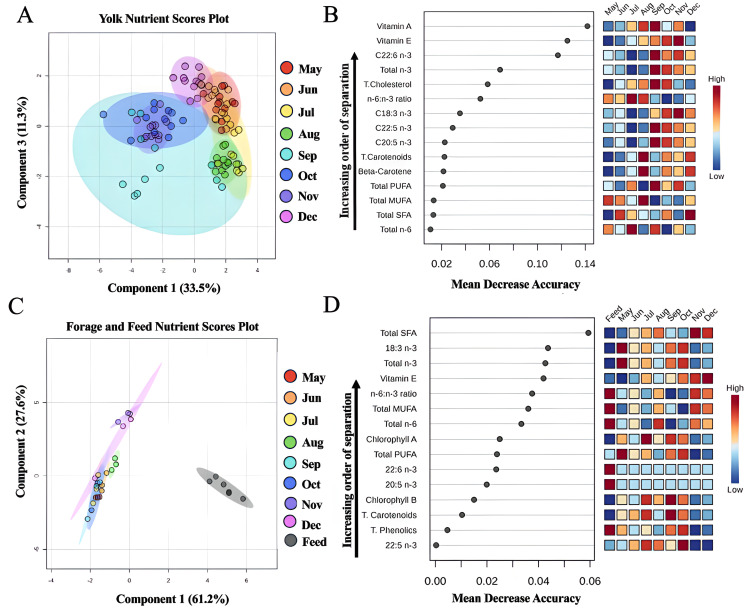
System nutrient structure. (A) sparse Partial Least Squares Discriminant Analysis (sPLS-DA) plot using egg nutrient parameters only showing separation and clusters based on month, with some overlaps. **(B)** Random Forest (RF) variable importance plot showing yolk nutrient parameters that differentiate between monthly collections. (C) sPLS-DA plot using forage and feed nutrient parameters only showing separation and clusters based on month, with some overlaps. **(D)** RF variable importance plot showing nutrient parameters that differentiate between monthly collections and layer hen feed. For sPLS-DA plots, ellipses are representative of 95% confidence interval regions. For RF plots, the y-axis represents nutrient parameters in order of importance for monthly classification (from top to bottom). The x-axis shows mean decrease accuracy, with a higher value indicating the importance of that phytochemical in predicting groups. Total SFA; total saturated fatty acids, total MUFA; total monounsaturated fatty acids, total PUFA; total polyunsaturated fatty acids, t. carotenoids; total carotenoids, total n-3; total omega-3 fatty acids, total n-6; total omega-6 fatty acids, t. phenolics; total phenolics.

The sPLS-DA and random forest analysis results are presented in Fig 7. (A) The sPLS-DA plot of yolk nutrients shows minimal separation between May to August and December, with noticeable differentiation observed during the fall months of September through November (S9 Table) (B) The forage and feed sPLS-DA scores plot indicate consistent overlap in forage nutrient profiles across all months, while feed nutrients exhibit distinct separation. (C) The random forest analysis highlights the importance of yolk nutrients in distinguishing individual months. Vitamins A and E emerged as the most discriminative variables, followed by DHA (C22:6 n-3), total n-3 fatty acids, and the n-6:n-3 ratio. These findings further confirm the separation of September through November from other seasons, driven by a lower n-6:n-3 ratio, higher levels of essential n-3 fatty acids, and elevated vitamin E and vitamin A concentrations during the fall months. (D) The forage and feed random forest analysis identified saturated fat and vitamin E as the most critical indicators of separation. The feed samples were characterized by a lower saturated fat profile and reduced vitamin E levels compared to forage. Vitamin E content was highest in the forage in December (S10 Table), meanwhile vitamin E was lowest in the eggs during the same month.

## Discussion

This study demonstrated that seasonal environmental fluctuations, including changes in weather, soil quality, and forage composition, had a measurable impact on the nutrient composition of pasture-raised eggs. We attribute changes in the fatty acid profiles, antioxidant concentrations, and some mineral contents to the availability and quality of forage, and hens’ adaptive feeding behaviors in response to environmental stressors. These findings underscore the dynamic nature of free-living pasture-raised systems, where nutrient outputs are closely tied to ecological variability across the grazing season, and are consistent with prior research in organic and pasture-based poultry systems showing that forage biodiversity, pasture management, and environmental inputs influence both animal welfare and the nutritional quality of eggs and meat [4–8].

Pasture-raising systems are shaped by numerous external influences on chicken habitats, identifying factors that impact dietary preferences, or the prediction of yolk nutrient deposition is challenging, emphasizing the need for this research. High forage consumption is typically expected to increase yolk levels of vitamin E, omega-3 PUFAs, carotenoids, and phenolics, as observed in grass-fed versus grain-fed systems [37,38]. However, pasture-raising systems are shaped by numerous external influences on chicken habitats and dietary patterns. Chickens are considered omnivores and cannot thrive on a forage-only diet; they prefer insect-based diets and are often consuming rocks and ground material to aid in their digestion [43,44]. Their opportunistic feeding habits make it difficult to accurately measure their intake while maintaining their pastured lifestyle, which may contribute to seasonal variations in yolk nutrient profiles.

The yolk n-6: n-3 ratio fluctuated across the season, ranging from 2.78 to 13.72. Eggs collected during the fall months (September–December) achieved the recommended 4:1 ratio, driven primarily by the increased n-3 content, as linoleic levels remained relatively stable [17]. The random forest analysis revealed that total n-3 and DHA levels in yolks did not align with the seasonal high and low omega-3 density observed in forage. Instead, yolks produced between September and November contained significantly higher amounts of n-3 PUFAs, vitamin E, and vitamin A, accompanied by the lowest n-6:n-3 ratio. We believe these improvements could be attributed to increased layer hen feed consumption, as feed was heavily supplemented with omega-3 fatty acids and vitamin A. Further, as the pasture sample collected were homogenous of the entire pasture, it is possible that the nutrient profile was not reflected of specific forage species that hens were consuming. The disconnect between forage nutrient density and yolk deposition could reflect a combination of factors including low actual forage intake, selective foraging behavior, nutrient loss during digestion, or differential metabolic prioritization of nutrients under varying environmental conditions.

Yolk CLA and BCFAs showed seasonal patterns influenced by diet, with CLA synthesis linked to linoleic acid levels [41,45,46]. Exposure to cattle in regenerative systems may account for the presence of uncommon BCFAs, such as C18:0-iso, which are typically found in cattle products and rarely detected in eggs [3,12,13,47,48]. Mineral content was relatively stable throughout the season, except for potassium, which spiked in the fall months. This increase likely reflects the layer hens’ higher consumption of feed rather than fresh forage during the colder months, as the feed contained a higher sodium concentration compared to forage. Additionally, higher consumption of feed is further supported by the sPLS-DA scores plot for yolk nutrients, which showed fall months clustering separately from earlier months and December. Similarly, forage and feed sPLS-DA plots demonstrated distinct groupings, suggesting feed became the primary driver of yolk nutrient changes during the fall. Additionally, weather deviations from seasonal norms—such as heavier or lighter rainfall observed in this system—may have further influenced forage quality and availability, thereby affecting hen foraging behavior and feeding preferences [49]. This pattern underscores the critical role of feed supplementation in meeting the nutritional needs of pasture-raised hens when environmental conditions limit forage availability.

Antioxidant deposition in egg yolks reflected the interplay between forage nutrient content and environmental stress. Vitamin E levels in yolks closely mirrored forage vitamin E levels throughout most of the season, except in December, when cold stress likely redirected vitamin E toward the hens’ metabolic needs rather than yolk deposition [50]. This inverse relationship may result from increased oxidative degradation of yolk vitamin E under cold stress, metabolic competition between vital and reproductive organs, or altered lipid transport that reduces delivery to the ovary despite high dietary intake [50,51]. Vitamin A content rose across the season, peaking in September before declining through December, a pattern that may be attributed to cold stress (<16 °C), as dropping temperatures prompt hens to prioritize heat generation over digestion and nutrient absorption, resulting in lower vitamin levels in eggs [50,51]. Carotenoid levels in yolks, however, remained stable even during colder months, showcasing the hens’ ability to maintain antioxidant deposition despite environmental challenges. Although carotenoid levels were preserved, yolk color—a key quality attribute linked to carotenoid concentration—did not meet consumer expectations. Consumer preferences align with the darkest values of the DSM Yolk Color Fan, which are associated with more nutrient-rich yolks [52]. However, yolks in this system fell below preferred values and were notably lighter than those of some cage-free eggs, which averaged a DSM score of 10.3 in a similar study [12,53,54]. It is also possible that differences in nutrient bioavailability and deposition efficiency, particularly under stress conditions, influenced which forage-derived compounds were absorbed and stored in yolk tissue. These findings highlight the importance of forage-derived antioxidants, particularly during the summer and early fall.

Additionally, seasonal changes in egg white protein quality, measured by Haugh units (HU), were closely tied to temperature fluctuations. Eggs produced in this system overall met the USDA AA quality standard (HU ≥ 72), except in August, when values dropped to Grade A [55,56]. Lower HU values were observed during the hot summer (July–August) and cold winter (November–December) months, aligning with environmental stressors outside the optimal laying temperature range of 19–22 °C [57]. The highest HU values occurred in May and September, when temperatures were within the thermoneutral zone, highlighting the role of temperature in albumen quality and freshness [58]. Based on our results, most eggs met the necessary criteria for sale under U.S. food laws. The only notable exception was albumen quality in August, where Haugh unit values fell into the USDA Grade A range rather than AA. While seasonal fluctuations in omega-3 levels were observed, these variations did not appear to impact regulatory compliance. However, such fluctuations could influence nutrient labeling or marketing claims related to omega-3 content. Further research could assess whether seasonal shifts impact classification for commercial sale.

Free-living systems carry the difficulty in controlling hen intake while adhering to pasture-raising principles, particularly regarding non-forage ingredients such as insects. Several insect species known to be nutrient-rich includingblack soldier fly larvae, crickets, mealworms, house flies, and maggots. These insectsare commonly found in Southern Ohio, particularly during the warmer months (May–September) when pasture-raised hens are actively foraging. They contain measurable amounts of omega-3 fatty acids and can also be rich in vitamin E, and carotenoids, though their nutritional profiles vary depending on life stage and diet [59,60]. While insect intake was not directly measured in this study, previous research suggests that insects, worms, and plants contribute to approximately 5–10% of the hens’ diet in pasture-based systems [59]. Insects likely comprise only a subset of that total, but given their nutrient density, even small amounts may meaningfully influence egg nutrient composition. The seasonal presence of these insects may help explain some of the trends in omega-3 and antioxidant levels observed in the eggs and points to an important area for future investigation.

As this study was conducted on a single farm in Southern Ohio, the results may not be generalizable to all pasture-raised systems. Regional variations in climate, forage composition, and management practices could influence egg nutrient profiles, necessitating multi-location studies to better understand these effects. Practical strategies to address the challenges of hen intake control, predation, and nutrient consistency will be critical for optimizing production. One example of such a strategy is targeted feed supplementation during periods of low forage quality. Regionally adapted approaches may help producers maintain consistent egg quality year-round. Additionally, the egg industry can benefit from characterizing seasonal nutrient shifts to improve year-round egg quality, offering consumers reliable access to nutrient-dense eggs.

## Conclusions

Future research should aim to evaluate these findings across different geographic regions and pasture management systems to determine their generalizability on a national scale. In this study, the months with the most favorable nutrient profile were in the fall, as nutrient profiles were at their peak due to seasonal shifts. However, the significant variation observed in nutrient profiles highlights the need for greater consistency in pasture-raised egg production. Refining grazing strategies and supplementation practices will be essential to ensure the year-round nutritional quality of pasture-raised eggs, thereby supporting sustainable agriculture while offering consumers reliable access to nutrient-rich foods.

## Supporting information

S1 Table Proximate analysis of the forage samples by month and the layer hen feed. (PDF)

S2 Table Fatty acid analysis of the forage samples by month and the layer hen feed (g per 100g). (PDF)

S3 Table Fatty acid analysis of the forage samples by month and the layer hen feed (percent of total fatty acids). (PDF)

S4 Table Antioxidant profile of the forage by month and the layer hen feed. (PDF)

S5 Table Antioxidant profile of the egg yolks by month. (PDF)

S6 Table Egg yolk fatty acids and cholesterol content by month (g of fatty acid per 100 g of fresh egg yolk). (PDF)

S7 Table Egg yolk fatty acids by month (% of total fatty acids). (PDF)

S8 Table Yolk mineral profile of the egg yolks by month. (PDF)

S9 Table Yolk sPLS-DA loading values. (PDF)

S10 Table Forage and Feed sPLS-DA loading values. (PDF)
