# Supplementary material for: Seasonal variation in egg nutrient composition under a pasture-based layer hen system: Implications for sustainable agriculture
Source: PLoS One. 2025 Sep 25;20(9):e0332411. doi: 10.1371/journal.pone.0332411 (PMC12463277; doi:10.1371/journal.pone.0332411)
Supplement: S1 Table — (PDF) [file pone.0332411.s001.pdf]

Table S1. Proximate analysis of the forage samples by month and the layer hen feed[1]

Parameter	May	Jun	Jul	Aug	Sept	Oct	Nov	Dec	*p*-value[2]	Layer Hen Feed
% Moisture	86.20 ± 1.92 a	76.00 ± 3.41 b	75.53 ± 2.41 b	66.93 ± 2.72 c	78.30 ± 2.46 b	78.90 ± 1.57 b	77.47 ± 3.56 b	72.57 ± 0.93 bc	<0.001	11.68 ± 0.53
% Dry matter (DM)	13.80 ± 1.92 c	24.00 ± 3.41 b	24.47 ± 2.41 b	33.07 ± 2.72 a	21.70 ± 2.46 b	21.10 ± 1.57 b	22.53 ± 3.56 b	27.43 ± 0.93 ab	<0.001	88.35 ± 0.54
Crude protein (%DM)	16.37± 1.55 ab	12.80 ± 0.62 ab	17.30 ± 2.20 a	12.10 ± 0.87 b	14.10 ± 1.00 ab	17.40 ± 1.76 a	14.90 ± 2.29 ab	12.47 ± 2.04 b	0.003	18.02 ± 1.67
ADF (% DM)	36.07 ± 1.19 c	39.67 ± 1.64 bc	40.73 ± 2.84 abc	45.97 ± 0.64 a	43.80 ± 4.03 ab	29.80 ± 1.61 d	45.60 ± 0.79 a	41.63 ± 1.07 abc	<0.001	6.65 ± 0.38
NDF (% DM)	58.70 ± 1.73 b	62.97 ± 3.30 ab	61.50 ± 2.43 ab	68.10 ± 3.75 a	62.27 ± 3.96 ab	49.87 ± 3.32 c	63.20 ± 3.85 ab	66.2 ± 0.56 ab	<0.001	13.28 ± 1.15
Lignin (% DM)	4.87 ± 0.32 d	6.27 ± 1.25 bcd	6.40 ± 0.92 bcd	10.03 ± 1.89 ab	9.40 ± 2.13 abc	5.57 ± 1.53 cd	9.40 ± 2.13 a	10.00 ± 1.22 ab	<0.001	2.32 ± 0.44
Starch (% DM)	0.37 ± 0.29	1.10 ± 0.26	1.30 ± 0.56	0.20 ± 0.00	0.50 ± 0.52	2.57 ± 2.76	1.30 ± 0.40	0.47 ± 0.25	0.183	38.82 ± 4.56
Crude fat (% DM)	3.27 ± 0.29	2.83 ± 0.21	3.37 ± 0.55	2.47 ± 0.47	3.33 ± 0.21	3.40 ± 0.53	2.43 ± 0.15	2.80 ± 0.46	0.024	4.50 ± 0.37
Ash (% DM)	11.26 ± 1.94 ab	15.14 ± 4.59 a	11.24 ± 1.16 ab	9.15 ± 1.66 b	9.35 ± 0.73 b	10.04 ± 0.26 ab	8.77 ± 1.30 b	7.76 ± 0.38 b	0.011	12.76 ± 1.36
TDN (% DM)	57.00 ± 2.00 ab	47.33 ± 5.13 bc	54.33 ± 2.08 abc	47.00 ± 3.46 c	51.33 ± 3.21 bc	61.00 ± 2.65 a	48.67 ± 2.89 bc	49.33 ± 3.21 bc	0.018	75.83 ± 1.72
ME (mcal/kg)	2.19 ± 0.08 ab	1.74 ± 0.21 cd	2.09 ± 0.12 abc	1.67 ± 0.13 d	1.91 ± 0.15 bcd	2.35 ± 0.12 a	1.78 ± 0.16 bcd	1.79 ± 0.16 bcd	<0.001	3.02 ± 0.07
Calcium (% DM)	0.42 ± 0.09 bc	0.33 ± 0.02 c	0.69 ± 0.14 a	0.54 ± 0.08 abc	0.71 ± 0.13 a	0.65 ± 0.02 ab	0.72 ± 0.10 a	0.57 ± 0.06 abc	0.001	3.12 ± 0.76
Phosphorus (% DM)	0.29 ± 0.01 a	0.19 ± 0.01 b	0.30 ± 0.04 a	0.17 ± 0.01 b	0.24 ± 0.03 ab	0.30 ± 0.02 a	0.23 ± 0.06 ab	0.22 ± 0.01 ab	<0.001	0.74 ± 0.11
Magnesium (% DM)	0.18 ± 0.03 b	0.16 ± 0.01 b	0.27 ± 0.02 b	0.22 ± 0.04 b	0.27 ± 0.03 b	0.40 ± 0.09 a	0.26 ± 0.05 b	0.21 ± 0.02 b	<0.001	0.25 ± 0.03
Potassium (% DM)	2.91 ± 0.42 a	1.10 ± 0.17 b	2.90 ± 0.49 a	1.09 ± 0.08 b	1.61 ± 0.34 b	2.83 ± 0.09 a	1.11 ± 0.66 b	0.83 ± 0.23 b	<0.001	0.75 ± 0.13
Sodium (% DM)	0.01 ± 0.01bc	0.04 ± 0.01a	0.02 ± 0.01 b	0.02 ± 0.01 bc	0.01 ± 0.01c	0.02 ± 0.01bc	0.01 ± 0.01 bc	0.01 ± 0.01bc	<0.001	0.24 ± 0.06
Sulfur (% DM)	0.30 ± 0.04 a	0.15 ± 0.07 b	0.28 ± 0.05 a	0.12 ± 0.02 b	0.2 ± 0.06 ab	0.31 ± 0.04 a	0.14 ± 0.03 b	0.23 ± 0.01 ab	<0.001	0.28 ± 0.05
Chloride (% DM)	0.78 ± 0.21 b	0.68 ± 0.21 b	0.88 ± 0.36 b	0.65 ± 0.09 ab	0.89 ± 0.11 b	1.23 ± 0.13 a	0.59 ± 0.24 ab	0.52 ± 0.13 b	0.015	0.44 ± 0.03
Iron (ppm)	109.00 ± 56.00 b	901.00 ± 466.00 a	160.00 ± 51.00 b	526.00 ± 118.00 ab	196.00 ± 140.50 b	49.00 ± 22.00 b	409.00 ± 134.50 ab	309.00 ± 126.00 ab	0.010	353.67 ± 104.25
Zinc (ppm)	3.00 ± 0.50	8.00 ± 1.50	8.00 ± 2.00	7.00 ± 1.50	7.00 ± 0.50	6.00 ± 1.50	7.00 ± 1.00	7.00 ± 0.50	0.170	115.00 ± 19.26
Copper (ppm)	1.00 ± 0.00	1.50 ± 0.50	2.00 ± 0.00	2.00 ± 0.50	2.00 ± 0.00	2.00 ± 0.50	2.00 ± 0.50	2.00 ± 0.50	0.061	21.83 ± 4.36
Manganese (ppm)	10.00 ± 3.50 c	95.00 ± 21.50 a	42.00 ± 14.00 abc	60.00 ± 12.50 ab	31.00 ± 11.50 bc	24.00 ± 8.50 c	47.00 ± 17.00 abc	73.00 ± 8.50 ab	0.012	106.50 ± 25.25
Molybdenum (ppm)	NA ± NA	0.50 ± 0.15	0.40 ± 0.15	0.40 ± 0.25	NA ± NA	NA ± NA	0.40 ± 0.10	0.30 ± 0.05	0.262	2.15 ± 1.11

[1]Means ± standard deviation (n = 3 forage per month, n = 6 layer hen feed samples) [2]Results of one-way ANOVA to compare forage by date. a-e, Means within a row for forage samples with different letters significantly differ ($p < 0.05$). DM, dry matter; ADF, acid detergent fiber; NDF, neutral detergent fiber; TDN, total digestible nutrients; ME, metabolizable energy.
