# Supplementary material for: Seasonal variation in egg nutrient composition under a pasture-based layer hen system: Implications for sustainable agriculture
Source: PLoS One. 2025 Sep 25;20(9):e0332411. doi: 10.1371/journal.pone.0332411 (PMC12463277; doi:10.1371/journal.pone.0332411)
Supplement: S2 Table — (PDF) [file pone.0332411.s002.pdf]

Table S2. Fatty acid analysis of the forage samples by month and the layer hen feed (g per 100g)[1]

Fatty Acid	Carbon Number	May	Jun	Jul	Aug	Sept	Oct	Nov	Dec	*p*-value[2]	Layer Hen Feed
Caprylic	8:0	0.025 ± 0.004 a	0.041 ± 0.002 a	0.071± 0.011 a	0.070 ± 0.008 a	0.057 ± 0.014 a	0.056 ± 0.011 a	0.057 ± 0.007 a	0.062 ± 0.122 a	0.024	0.168 ± 0.070
Capric	10:0	0.005 ± 0.001	0.008 ± 0.009	0.007± 0.002	0.007 ± 0.004	0.005± 0.001	0.005 ± 0.001	0.005 ± 0.001	0.034 ± 0.073	0.131	0.014 ± 0.004
Undecanoic	11:0	0.001± 0.001	0.001 ± 0.001	0.001± 0.001	0.001 ± 0.001	0.001± 0.001	0.001 ± 0.001	0.001 ± 0.001	0.001 ± 0.003	0.818	0.002 ± 0.001
Lauric	12:0	0.013± 0.003 a	0.047 ± 0.003 a	0.046 ± 0.023 a	0.035 ± 0.004 a	0.038 ± 0.004 a	0.037 ± 0.005 a	0.023 ± 0.005 a	0.035 ± 0.051 a	0.037	0.025 ± 0.010
Tridecanoic	13:0	LOD	0.001 ± 0.001	LOD	LOD	LOD	LOD	LOD	LOD	0.362	0.002 ± 0.001
Myristic	14:0	0.038 ± 0.012	0.065 ± 0.027.	0.054 ± 0.030	0.064 ± 0.007	0.033 ± 0.001	0.052 ± 0.006	0.067 ± 0.024	0.059 ± 0.064	0.056	1.104 ± 0.435
Myristoleic	14:1	LOD	LOD	LOD	LOD	LOD	LOD	LOD	LOD	NA	LOD
Pentadecanoic	15:0	0.015 ± 0.007 b	0.022 ± 0.003 ab	0.015 ± 0.002 b	0.042 ± 0.005 a	0.007 ± 0.001 b	0.008 ± 0.001 b	0.017 ± 0.010 ab	0.006 ± 0.015 b	0.034	0.086 ± 0.033
Palmitic	16:0	1.136 ± 0.500	1.533 ± 0.169	2.612 ± 0.344.	2.554 ± 0.213	1.425 ± 0.263.	1.722 ± 0.205	1.223 ± 0.562	0.643 ± 2.714	0.113	21.411 ± 9.568
Palmiteladic	16:1 n-9t	LOD	LOD	LOD	LOD	LOD	LOD	LOD	LOD	NA	LOD
Palmitoleic	16:1 n-7	0.103 ± 0.038	0.050 ± 0.005	0.117 ± 0.021	0.055 ± 0.006	0.041 ± 0.016	0.062 ± 0.022	0.016 ± 0.009	0.011 ± 0.125	0.060	0.082 ± 0.038
	16:1 n-9	0.018 ± 0.009 c	0.059 ± 0.011 abc	0.048 ± 0.003 abc	0.127 ± 0.014 a	0.029 ± 0.003 bc	0.027 ± 0.029 abc	0.115 ± 0.027 abc	0.085 ± 0.078 ab	0.012	1.635 ± 0.779
Heptadecanoic	17:0	0.015 ± 0.009	0.028 ± 0.003	0.045 ± 0.007	0.057 ± 0.004	0.024 ± 0.001	0.023 ± 0.003	0.028 ± 0.011	0.015 ± 0.030	0.123	0.177 ± 0.070
c10-heptadecanoic	17:1	LOD	LOD	LOD	LOD	LOD	LOD	LOD	LOD	NA	LOD
Stearic	18:0	0.166 ± 0.078 a	0.222 ± 0.030 a	0.459 ± 0.055 a	0.529 ± 0.054 a	0.296 ± 0.027 a	0.349 ± 0.029 a	0.399 ± 0.097 a	0.223 ± 0.539 a	0.049	3.461 ± 1.365
Eladic	18:1 n-9t	LOD	LOD	LOD	LOD	LOD	LOD	LOD	LOD	NA	LOD
Oleic	18:1 n-9	0.203 ± 0.080 a	0.351 ± 0.041 a	0.560 ± 0.051 a	0.872 ± 0.109 a	0.393 ± 0.032 a	0.461 ± 0.114 a	0.496 ± 0.180 a	0.643 ± 0.768 a	0.007	30.802 ± 16.904
	18:1 n-11	0.254 ± 0.190 b	0.467 ± 0.086 b	0.227 ± 0.214 b	1.247 ± 0.157 a	0.171 ± 0.026 b	0.057 ± 0.076 b	0.410 ± 0.162 b	0.248 ± 0.228 b	0.037	9.440 ± 4.874
Linoleic	18:2 n-6	1.528 ± 0.579	1.564 ± 0.064	2.219 ± 0.310	2.917 ± 0.100	1.282 ± 0.316	1.695 ± 0.317	1.089 ± 0.160	0.799 ± 2.056	0.082	84.986 ± 41.376
ALA	18:3 n-3	4.256 ± 1.613	3.744 ± 0.252	6.681 ± 1.20	3.987 ± 0.561	3.860 ± 1.957	4.899 ± 1.670	0.940 ± 0.387	0.761 ± 8.744	0.117	5.530 ± 2.200
GLA	18:3 n-6	LOD	LOD	LOD	LOD	LOD	LOD	LOD	LOD	NA	LOD
Arachidic	20:0	0.107 ± 0.063	0.115 ± 0.014	0.146 ± 0.047	0.141 ± 0.014	0.074 ± 0.010	0.075 ± 0.015	0.082 ± 0.021	0.062 ± 0.196	0.122	0.769 ± 0.471
Eicosenoic	20:1 n-9	0.022 ± 0.006	0.015 ± 0.002	0.026 ± 0.006	0.033 ± 0.003	0.023 ± 0.002	0.022 ± 0.003	0.018 ± 0.004	0.017 ± 0.037	0.080	0.833 ± 0.512
Eicosedienoic	20:2 n-6	0.010 ± 0.002 ab	0.007 ± 0.001 b	0.014 ± 0.006 ab	0.018 ± 0.003 a	0.006 ± 0.002 b	0.008 ± 0.004 ab	0.004 ± 0.001 b	0.005 ± 0.002 b	0.041	0.136 ± 0.110
Eicosatrenoic	20:3 n-3	0.018 ± 0.006	0.013 ± 0.001	0.030 ± 0.002	0.021 ± 0.005	0.020 ± 0.005	0.023 ± 0.002	0.023 ± 0.004	0.021 ± 0.037	0.075	0.070 ± 0.028
DGLA	20:3 n-6	LOD	LOD	LOD	LOD	LOD	LOD	LOD	LOD	NA	LOD
Mead	20:9 n-9	LOD	LOD	LOD	LOD	LOD	LOD	LOD	LOD	NA	LOD
Arachidonic	20:4 n-6	LOD	0.015 ± 0.004	0.035 ± 0.006	0.032 ± 0.005	0.023 ± 0.008	0.022 ± 0.003	0.028 ± 0.009	0.026 ± 0.044	0.078	0.248 ± 0.108
EPA	20:5 n-3	LOD	LOD	LOD	LOD	LOD	LOD	LOD	LOD	NA	1.495 ± 0.903
Behenic	22:0	0.144 ± 0.066	0.142 ± 0.014	0.273 ± 0.017	0.260 ± 0.028	0.175 ± 0.039	0.162 ± 0.024	0.189 ± 0.024	0.157 ± 0.343	0.124	0.738 ± 0.299
DTA	22:4 n-6	LOD	LOD	LOD	LOD	LOD	LOD	LOD	LOD	NA	LOD
DPA	22:5 n-3	LOD	LOD	LOD	LOD	LOD	LOD	LOD	LOD	NA	2.612 ± 1.952
	22:5 n-6	0.068 ± 0.003 b	0.056 ± 0.019 b	0.098 ± 0.005 ab	0.132 ± 0.030 a	0.080 ± 0.003 ab	0.082 ± 0.010 ab	LOD	LOD	0.035	0.377 ± 0.165
DHA	22:6 n-3	LOD	LOD	LOD	LOD	LOD	LOD	LOD	LOD	NA	1.513 ± 0.856
Lignoceric	24:0	0.147 ± 0.061 a	0.131 ± 0.013 a	0.203 ± 0.089 a	0.196 ± 0.025 a	0.063 ± 0.019 a	0.057 ± 0.011 a	0.086 ± 0.016 a	0.102 ± 0.179 a	0.034	0.489 ± 0.361
Total SFA		1.798 ± 0.795	2.307 ± 0.204	3.908 ± 0.582	3.95 ± 0.311	2.348 ± 0.227	2.647 ± 0.235	2.292 ± 0.709	1.308 ± 4.284	0.131	28.111 ± 12.833

Total MUFA	0.600 ± 0.322	0.993 ± 0.109	0.975 ± 0.178	2.142 ± 0.149	0.687 ± 0.050	0.625 ± 0.198	1.012 ± 0.348	1.000 ± 1.232	0.088	40.898 ± 26.029
Total PUFA	5.875 ± 2.235	5.374 ± 0.225	9.087 ± 1.503	6.975 ± 0.703	5.212 ± 2.271	6.724 ± 1.966	2.252 ± 0.474	1.550 ± 10.854	0.155	95.122 ± 50.191
Total n-6	1.601 ± 0.616	1.648 ± 0.084	2.376 ± 0.304	2.967 ± 0.139	1.324 ± 0.314	1.802 ± 0.296	1.122 ± 0.167	0.823 ± 2.102	0.084	81.700 ± 48.876
Total n-3	4.275 ± 1.619	3.757 ± 0.253	6.711 ± 1.198	4.008 ± 0.565	3.888 ± 1.958	4.922 ± 1.669	0.970 ± 0.387	0.782 ± 8.780	0.117	13.422 ± 5.305
n-6:n-3 ratio	0.341 ± 0.033	0.420 ± 0.041	0.341 ± 0.020	0.740 ± 0.077	0.341 ± 0.055	0.348 ± 0.043	1.322 ± 0.345	0.981 ± 0.652	0.051	6.805 ± 3.394
Total OCFA	0.031 ± 0.015	0.051 ± 0.005	0.060 ± 0.009	0.099 ± 0.010	0.032 ± 0.002	0.031 ± 0.003	0.046 ± 0.019	0.021 ± 0.048	0.096	0.259 ± 0.110
Total FA	8.273 ± 3.352	8.674 ± 0.537	14.801 ± 1.847	13.328 ± 0.996	8.278 ± 2.533	9.839 ± 2.320	6.237 ± 1.175	3.652 ± 16.268	0.121	164.131 ± 88.932

[1]Means ± standard deviation n = 3 forage replicates per month and layer hen feed n=6 [2]Results of one-way ANOVA. a-e, Means within a row with different letters significantly differ $p < 0.05$. SFA, saturated fatty acids; MUFA, monounsaturated fatty acids, PUFA, polyunsaturated fatty acids; OCFA, odd-chain fatty acids; FA, fatty acids.
