# Supplementary material for: Seasonal variation in egg nutrient composition under a pasture-based layer hen system: Implications for sustainable agriculture
Source: PLoS One. 2025 Sep 25;20(9):e0332411. doi: 10.1371/journal.pone.0332411 (PMC12463277; doi:10.1371/journal.pone.0332411)
Supplement: S3 Table — (PDF) [file pone.0332411.s003.pdf]

Table S3. Fatty acid analysis of the forage samples by date and the layer hen feed (percent of total fatty acids)[1]

Fatty Acid	Carbon Number	May	Jun	Jul	Aug	Sept	Oct	Nov	Dec	*p*-value[2]	Layer Hen Feed
Caprylic	8:0	0.289 ± 0.061 c	0.467 ± 0.006 bc	0.481 ± 0.159 bc	0.551 ± 0.050 bc	0.763 ± 0.275 abc	0.580 ± 0.186 bc	1.057 ± 0.176 ab	1.262 ± 0.496 a	0.018	0.175 ± 0.048
Capric	10:0	0.051 ± 0.025 b	0.084 ± 0.112 ab	0.058 ± 0.012 b	0.047 ± 0.027 b	0.066 ± 0.022 b	0.051 ± 0.020 b	0.097 ± 0.018 b	0.425 ± 0.401 a	0.038	0.010 ± 0.004
Undecanoic	11:0	0.008 ± 0.001 b	0.013 ± 0.002 ab	0.010 ± 0.005 ab	0.007 ± 0.003 b	0.009 ± 0.002 b	0.006 ± 0.002 b	0.014 ± 0.003 ab	0.017 ± 0.003 a	0.028	0.002 ± 0.001
Lauric	12:0	0.131 ± 0.025 b	0.506 ± 0.048 ab	0.298 ± 0.127 ab	0.249 ± 0.042 ab	0.453 ± 0.142 ab	0.387 ± 0.114 ab	0.506 ± 0.088 ab	0.575 ± 0.349 a	0.040	0.016 ± 0.004
Tridecanoic	13:0	LOD	0.006 ± 0.004 a	LOD	0.001 ± 0.001 b	LOD	LOD	LOD	LOD	0.027	0.002 ± 0.001
Myristic	14:0	0.327 ± 0.084 b	0.697 ± 0.347 ab	0.347 ± 0.173 b	0.524 ± 0.064 ab	0.399 ± 0.097 b	0.436 ± 0.073 b	1.630 ± 0.399 a	1.000 ± 0.629 ab	0.026	0.722 ± 0.217
Myristoleic	14:1	LOD	LOD	LOD	LOD	LOD	LOD	LOD	LOD	NA	LOD
Pentadecanoic	15:0	0.152 ± 0.030 bc	0.249 ± 0.013 ab	0.114 ± 0.017 c	0.300 ± 0.033 a	0.085 ± 0.041 c	0.076 ± 0.018 c	0.351 ± 0.116 a	0.136 ± 0.032 bc	0.005	0.057 ± 0.021
Palmitic	16:0	14.045 ± 0.363 b	16.756 ± 1.411 ab	18.061 ± 1.279 ab	20.129 ± 0.571 ab	17.212 ± 1.486 ab	16.759 ± 1.653 ab	19.604 ± 5.46 a	16.591 ± 0.936 ab	0.017	13.049 ± 0.879
Palmiteladic	16:1 n-9t	LOD	LOD	LOD	LOD	LOD	LOD	LOD	LOD	NA	LOD
Palmitoleic	16:1 n-7	1.158 ± 0.050 a	0.577 ± 0.085 bc	0.764 ± 0.073 b	0.448 ± 0.031 bc	0.507 ± 0.028 bc	0.628 ± 0.068 bc	0.260 ± 0.096 c	0.320 ± 0.257 bc	0.014	0.050 ± 0.005
	16:1 n-9	0.220 ± 0.014 c	0.680 ± 0.091 bc	0.327 ± 0.064 c	1.043 ± 0.131 bc	0.388 ± 0.101 c	0.188 ± 0.294 c	1.954 ± 0.183 a	1.616 ± 0.865 ab	0.010	1.006 ± 0.189
Heptadecanoic	17:0	0.189 ± 0.024 c	0.319 ± 0.023 bc	0.286 ± 0.021 bc	0.408 ± 0.021 ab	0.288 ± 0.054 bc	0.237 ± 0.058 c	0.483 ± 0.094 a	0.374 ± 0.105 bc	0.013	0.109 ± 0.012
c10-heptadecanoic	17:1	LOD	LOD	LOD	LOD	LOD	LOD	LOD	LOD	NA	LOD
Stearic	18:0	2.003 ± 0.101 c	2.563 ± 0.184 c	3.357 ± 0.262 c	3.728 ± 0.251 bc	3.922 ± 0.906 bc	3.039 ± 0.575 c	6.864 ± 0.496 a	6.103 ± 1.269 ab	0.010	2.180 ± 0.342
Eladic	18:1 n-9t	LOD	LOD	LOD	LOD	LOD	LOD	LOD	LOD	NA	LOD
Oleic	18:1 n-9	2.497 ± 0.074 c	4.16 ± 0.261 bc	3.578 ± 0.811 bc	6.373 ± 0.458 abc	5.285 ± 1.163 bc	4.594 ± 0.196 bc	12.134 ± 2.992 ab	14.636 ± 5.969 a	0.006	17.793 ± 1.634
	18:1 n-11	3.073 ± 0.850 b	5.037 ± 0.870 ab	1.452 ± 1.310 b	9.776 ± 1.628 a	1.798 ± 0.283 b	0.589 ± 0.464 b	10.030 ± 2.703 a	2.882 ± 2.620 b	0.007	5.572 ± 0.577
Linoleic	18:2 n-6	17.021 ± 0.827 ab	18.511 ± 1.065 ab	15.243 ± 0.304 b	21.884 ± 0.885 a	15.490 ± 0.642 b	15.935 ± 1.057 ab	19.842 ± 2.756 ab	21.613 ± 4.190 ab	0.044	50.570 ± 1.517
ALA	18:3 n-3	51.447 ± 1.524 a	44.399 ± 1.974 abc	48.147 ± 3.484 abc	29.914 ± 2.009 bcd	46.635 ± 6.275 ab	50.547 ± 3.937 a	15.078 ± 3.279 d	22.263 ± 18.106 cd	0.018	3.540 ± 0.780
GLA	18:3 n-6	LOD	LOD	LOD	LOD	LOD	LOD	LOD	LOD	NA	LOD
Arachidic	20:0	1.382 ± 0.164 a	1.348 ± 0.144 ab	0.930 ± 0.243 ab	1.123 ± 0.053 ab	0.989 ± 0.256 ab	0.642 ± 0.211 b	1.609 ± 0.224 a	1.166 ± 0.455 ab	0.031	0.424 ± 0.088
Eicosenoic	20:1 n-9	0.267 ± 0.023 ab	0.184 ± 0.016 b	0.166 ± 0.065 b	0.235 ± 0.011 ab	0.300 ± 0.071 ab	0.193 ± 0.028 b	0.378 ± 0.056 ab	0.475 ± 0.128 a	0.038	0.466 ± 0.085
Eicosedienoic	20:2 n-6	0.101 ± 0.017	0.076 ± 0.008	0.117 ± 0.046	0.132 ± 0.015	0.077 ± 0.042	0.086 ± 0.020	0.069 ± 0.054	0.023 ± 0.044	0.260	0.071 ± 0.037
Eicosatrenoic	20:3 n-3	0.221 ± 0.021 ab	0.160 ± 0.009 b	0.203 ± 0.042 ab	0.159 ± 0.020 b	0.252 ± 0.090 ab	0.234 ± 0.051 ab	0.483 ± 0.109 a	0.443 ± 0.186 a	0.028	0.058 ± 0.016
DGLA	20:3 n-6	LOD	LOD	LOD	LOD	LOD	LOD	LOD	LOD	NA	LOD
Mead	20:9 n-9	LOD	LOD	LOD	LOD	LOD	LOD	LOD	LOD	NA	LOD
Arachidonic	20:4 n-6	LOD	0.167 ± 0.026 b	0.238 ± 0.090 ab	0.240 ± 0.021 ab	0.312 ± 0.136 ab	0.207 ± 0.126 b	0.587 ± 0.113 a	0.675 ± 0.222 a	0.016	0.155 ± 0.023
EPA	20:5 n-3	LOD	LOD	LOD	LOD	LOD	LOD	LOD	LOD	NA	0.859 ± 0.199
Behenic	22:00	1.872 ± 0.213 bc	1.729 ± 0.102 c	1.843 ± 0.392 bc	2.052 ± 0.105 abc	2.355 ± 0.769 abc	1.668 ± 0.484 c	3.352 ± 0.510 ab	3.982 ± 1.140 a	0.046	0.464 ± 0.079
DTA	22:4 n-6	LOD	LOD	LOD	LOD	LOD	LOD	LOD	LOD	NA	LOD
DPA	22:5 n-3	LOD	LOD	LOD	LOD	LOD	LOD	LOD	LOD	NA	1.374 ± 0.547
	22:5 n-6	0.510 ± 0.384	0.606 ± 0.207	0.667 ± 0.060	0.832 ± 0.570	0.665 ± 0.525	0.848 ± 0.226	LOD	LOD	0.149	0.236 ± 0.040
DHA	22:6 n-3	LOD	LOD	LOD	LOD	LOD	LOD	LOD	LOD	NA	0.893 ± 0.230

Lignoceric	24:0	1.678 ± 0.109 ab	1.592 ± 0.087 ab	1.300 ± 0.499 ab	1.496 ± 0.192 ab	0.850 ± 0.313 ab	0.530 ± 0.166 b	1.612 ± 0.163 ab	1.393 ± 0.833 a	0.043	0.255 ± 0.101
Total SFA		22.036 ± 0.563 c	26.594 ± 0.730 bc	27.497 ± 2.555 bc	30.767 ± 0.594 abc	28.087 ± 4.015 bc	24.414 ± 3.558 bc	36.756 ± 4.371 a	34.694 ± 4.34 ab	0.013	17.375 ± 1.392
Total MUFA		7.257 ± 0.785 bc	10.987 ± 0.839 bc	7.584 ± 1.149 bc	17.569 ± 1.591 abc	8.290 ± 1.586 bc	6.350 ± 0.372 c	24.772 ± 5.831 a	19.965 ± 9.31 ab	0.009	24.888 ± 1.728
Total PUFA		71.015 ± 1.194 a	62.325 ± 1.523 abc	64.919 ± 3.704 abc	52.333 ± 1.331 bcd	63.623 ± 5.601 ab	69.374 ± 3.255 a	38.84 ± 1.643 d	45.34 ± 13.65 cd	0.013	57.738 ± 0.662
Total n-6		17.614 ± 1.218	19.209 ± 1.181	16.494 ± 0.224	22.430 ± 0.784	15.997 ± 0.954	17.233 ± 1.312	20.561 ± 2.886	22.455 ± 4.359	0.071	51.032 ± 1.502
Total n-3		51.667 ± 1.546 a	44.566 ± 1.979 abc	48.425 ± 3.48 abc	30.073 ± 2.03 bcd	46.971 ± 6.227 ab	50.781 ± 3.886 a	15.637 ± 3.209 d	22.885 ± 18.009 cd	0.018	6.705 ± 1.055
n-6:n-3 ratio		0.341 ± 0.033	0.420 ± 0.041	0.341 ± 0.020	0.740 ± 0.077	0.341 ± 0.055	0.348 ± 0.043	1.322 ± 0.345	0.981 ± 0.652	0.051	7.791 ± 1.390
Total OCFA		0.369 ± 0.038 c	0.592 ± 0.027 bc	0.416 ± 0.033 c	0.718 ± 0.053 ab	0.382 ± 0.097 c	0.322 ± 0.077 c	0.847 ± 0.206 a	0.529 ± 0.134 bc	0.008	0.168 ± 0.034

[1]Means ± standard deviation (n = 3 forage replicates per month, n = 6 layer hen feed samples) [2]Results of one-way ANOVA. a-e, Means within a row with different letters significantly differ $p < 0.05$. SFA, saturated fatty acids; MUFA, monounsaturated fatty acids, PUFA, polyunsaturated fatty acids; OCFA, odd-chain fatty acids; FA, fatty acids.
