# Supplementary material for: Seasonal variation in egg nutrient composition under a pasture-based layer hen system: Implications for sustainable agriculture
Source: PLoS One. 2025 Sep 25;20(9):e0332411. doi: 10.1371/journal.pone.0332411 (PMC12463277; doi:10.1371/journal.pone.0332411)
Supplement: S4 Table — (PDF) [file pone.0332411.s004.pdf]

Table S4. Antioxidant profile of the forage by month and the layer hen feed[1]

Parameter	May	Jun	Jul	Aug	Sept	Oct	Nov	Dec	*p*-value[2]	Layer Hen Feed
Vitamin A (ng/g DM)	ND	ND	ND	ND	ND	ND	ND	ND	NA	10623.67 ± 767.76
Beta-carotene (ug/g DM)	NA	NA	NA	NA	NA	NA	3.16 ± 3.32	5.28 ± 5.47	NA	NA
Vitamin E (ug/g DM)	9.90 ± 1.48 c	28.10 ± 0.97 bc	108.72 ± 56.69 ab	29.78 ± 3.86 bc	53.22 ± 20.84 bc	103.40 ± 51.02 ab	104.17 ± 25.50 ab	158.94 ± 13.34 a	<0.001	11.21 ± 10.76
Chlorophyll a (ug/g DM)	2748.64 ± 195.42 a	1557.10 ± 270.13 abc	2327.67 ± 228.46 ab	1138.18 ± 166.5 bc	2614.71 ± 1121.41 ab	2278.5 ± 410.71 ab	347.74 ± 165.31 c	716.74 ± 778.80 c	<0.001	17.22 ± 5.30
Chlorophyll b (ug/g DM)	976.30 ± 71.32 a	556.26 ± 117.71 abc	781.41 ± 103.01 ab	493.34 ± 59.05 bc	927.66 ± 324.63 ab	791.32 ± 149.99 ab	149.22 ± 15.25 c	269.51 ± 223.06 c	<0.001	22.10 ± 8.25
Total carotenoids (ug/g DM)	765.92 ± 43.66 a	461.03 ± 94.35 abcd	626.32 ± 85.66 abc	270.47 ± 36.25 bcd	757.95 ± 378.49 ab	671.46 ± 113.89 abc	73.06 ± 5.80 d	219.65 ± 251.93 cd	0.001	14.47 ± 2.67
Total phenolic content (mg GAE/g DM)	4.143 ± 0.474 a	2.284 ± 0.606 bcd	2.019 ± 0.526 bcd	1.302 ± 0.272 cd	2.571 ± 0.554 abc	3.644 ± 0.735 ab	0.546 ± 0.471 d	1.606 ± 0.493 cd	0.008	0.91 ± 0.25

[1]Means ± standard deviation (n = 3 forage replicates per month, n = 6 layer hen feed samples) [2]Results of one-way ANOVA to compare forage by date. a-e, Means within a row for forage samples with different letters significantly differ ($p < 0.05$). DM, dry matter; ND, not detected; NA, value not determined for specific collection
