# Supplementary material for: Seasonal variation in egg nutrient composition under a pasture-based layer hen system: Implications for sustainable agriculture
Source: PLoS One. 2025 Sep 25;20(9):e0332411. doi: 10.1371/journal.pone.0332411 (PMC12463277; doi:10.1371/journal.pone.0332411)
Supplement: S5 Table — (PDF) [file pone.0332411.s005.pdf]

Table S5. Antioxidant profile of the egg yolks by month[1]

Parameter	May	Jun	Jul	Aug	Sept	Oct	Nov	Dec	*p*-value[2]
Vitamin A (ug/g FW)	4.04 ± 0.63 d	4.19 ± 0.38 d	6.97 ± 0.67 bc	9.03 ± 1.89 ab	10.80 ± 3.88 a	6.62 ± 1.97 c	7.77 ± 0.63 bc	2.85 ± 0.39 d	<0.001
Vitamin E (ug/g FW)	4.65 ± 6.01 e	14.90 ± 19.23 de	33.52 ± 18.06 d	55.12 ± 9.79 c	68.82 ± 13.02 bc	81.42 ± 17.57 b	118.06 ± 23.89 a	25.72 ± 6.90 d	<0.001
Total carotenoids (ug/g FW)	16.34 ± 7.95 c	20.64 ± 5.65 c	34.95 ± 11.74 abc	48.94 ± 12.19 a	29.92 ± 12.03 bc	44.39 ± 28.13 ab	39.59 ± 6.57 ab	49.69 ± 19.44 a	<0.001
Beta carotene (ug/g FW)	14.88 ± 7.34 c	18.85 ± 5.20 c	31.75 ± 11.03 abc	44.85 ± 10.99 a	27.37 ± 11.01 bc	40.75 ± 25.93 ab	36.25 ± 6.84 ab	45.23 ± 17.72 a	<0.001
Total phenolic content (mg GAE/g FW)	0.14 ± 0.02 ab	0.13 ± 0.02 ab	0.11 ± 0.01 b	0.14 ± 0.02 a	0.14 ± 0.02 a	0.14 ± 0.02 ab	0.13 ± 0.03 ab	0.14 ± 0.03 ab	0.019

[1]Means ± standard deviation (n = 24 eggs pooled into n = 12 replicates per month) [2]Results of one-way ANOVA. a-e, Means within a row with different letters significantly differ ($p < 0.05$). FW, fresh weight; GAE, gallic acid equivalents
