# Supplementary material for: Seasonal variation in egg nutrient composition under a pasture-based layer hen system: Implications for sustainable agriculture
Source: PLoS One. 2025 Sep 25;20(9):e0332411. doi: 10.1371/journal.pone.0332411 (PMC12463277; doi:10.1371/journal.pone.0332411)
Supplement: S6 Table — (PDF) [file pone.0332411.s006.pdf]

Table S6. Egg yolk fatty acids and cholesterol content by month (g of fatty acid per 100 g of fresh egg yolk)[1]

Fatty Acid	Carbon Number	May	Jun	Jul	Aug	Sept	Oct	Nov	Dec	*p*-value[2]
Caprylic	8:0	LOD	LOD	LOD	LOD	LOD	LOD	LOD	LOD	ND
Capric	10:0	0.001 ± 0.001 c	0.001 ± 0.001 bc	0.002 ± 0.001 ab	0.002 ± 0.001 a	0.001 ± 0.001	0.001 ± 0.001 c	0.001 ± 0.001 c	0.001 ± 0.001 c	<0.001
Undecanoic	11:0	LOD	0.001 ± 0.001 ab	0.001 ± 0.001 abc	0.001 ± 0.001 a	LOD	LOD	LOD	LOD	<0.001
Lauric	12:0	LOD	0.001 ± 0.001 b	0.001 ± 0.001 a	0.001 ± 0.001 a	0.001 ± 0.001 bc	LOD	LOD	LOD	<0.001
Tridecanoic	13:0	0.002 ± 0.001 ab	0.002 ± 0.001 ab	0.002 ± 0.001 a	0.002 ± 0.001 ab	0.002 ± 0.001 ab	0.001 ± 0.001 c	0.002 ± 0.001 b	0.001 ± 0.001 c	<0.001
Myristic	14:0	0.061 ± 0.014 ab	0.065 ± 0.019 a	0.050 ± 0.009 abc	0.047 ± 0.009 bc	0.057 ± 0.014 ab	0.041 ± 0.013 c	0.065 ± 0.008 a	0.036 ± 0.007 c	<0.001
Myristoleic	14:1	0.010 ± 0.003 cd	0.015 ± 0.004 ab	0.008 ± 0.004 d	0.011 ± 0.003 bcd	0.014 ± 0.005 abc	0.013 ± 0.005 abcd	0.016 ± 0.003 a	0.008 ± 0.002 d	<0.001
Pentadecanoic	15:0	0.014 ± 0.002 ab	0.012 ± 0.004 abc	0.011 ± 0.002 bc	0.010 ± 0.002 c	0.015 ± 0.003 a	0.009 ± 0.003 c	0.015 ± 0.003 a	0.009 ± 0.002 c	<0.001
Palmitic	16:0	4.939 ± 1.097 a	3.700 ± 0.478 bcd	3.987 ± 0.388 bc	3.405 ± 0.385 cd	4.328 ± 0.756 ab	2.956 ± 0.726 d	4.120 ± 0.650 abc	3.052 ± 0.507 d	<0.001
Palmiteladic	16:1 n-9t	0.009 ± 0.002 bc	0.007 ± 0.002 c	0.007 ± 0.002 c	0.007 ± 0.001 c	0.011 ± 0.004 ab	0.009 ± 0.002 bc	0.013 ± 0.002 a	0.007 ± 0.002 c	<0.001
Palmitoleic	16:1 n-7	0.146 ± 0.035 a	0.078 ± 0.015 bcd	0.093 ± 0.020 b	0.059 ± 0.012 d	0.100 ± 0.027 b	0.064 ± 0.019 cd	0.090 ± 0.018 bc	0.074 ± 0.014 bcd	<0.001
	16:1 n-9	0.492 ± 0.094 abcd	0.500 ± 0.097 abc	0.362 ± 0.103 cd	0.429 ± 0.069 bcd	0.556 ± 0.159 ab	0.465 ± 0.143 bcd	0.626 ± 0.099 a	0.360 ± 0.084 d	<0.001
Heptadecanoic	17:0	0.039 ± 0.011 ab	0.030 ± 0.003 bc	0.032 ± 0.007 bc	0.024 ± 0.004 c	0.046 ± 0.014 a	0.028 ± 0.004 c	0.042 ± 0.008 a	0.030 ± 0.006 bc	<0.001
c10-heptadecanoic	17:1	LOD	LOD	LOD	LOD	LOD	LOD	LOD	LOD	ND
Stearic	18:0	0.039 ± 0.008 ab	0.030 ± 0.009 bc	0.032 ± 0.005 bc	0.024 ± 0.003 c	0.044 ± 0.010 a	0.029 ± 0.007 c	0.042 ± 0.006 a	0.030 ± 0.005 bc	<0.001
Eladic	18:1 n-9t	0.034 ± 0.008 abc	0.029 ± 0.006 bcd	0.026 ± 0.007 cd	0.019 ± 0.004 d	0.038 ± 0.017 ab	0.036 ± 0.013 abc	0.042 ± 0.009 a	0.030 ± 0.005 abcd	<0.001
Oleic	18:1 n-9	8.466 ± 1.589 a	5.424 ± 0.565 bc	6.398 ± 0.653 b	5.657 ± 0.730 b	6.235 ± 1.192 b	4.217 ± 1.355 c	6.077 ± 0.758 b	4.331 ± 0.713 c	<0.001
	18:1 n-11	0.323 ± 0.060 ab	0.235 ± 0.030 cd	0.189 ± 0.040 cd	0.180 ± 0.029 d	0.374 ± 0.137 a	0.270 ± 0.074 bc	0.389 ± 0.058 a	0.236 ± 0.048 cd	<0.001
Linoleic	18:2 n-6	3.405 ± 0.995 a	2.016 ± 0.540 bc	3.034 ± 1.176 a	1.892 ± 0.311 bc	2.666 ± 0.658 ab	1.475 ± 0.560 c	2.687 ± 0.467 ab	1.604 ± 0.272 c	<0.001
ALA	18:3 n-3	0.130 ± 0.028 bcd	0.108 ± 0.029 cde	0.077 ± 0.019 de	0.076 ± 0.014 e	0.159 ± 0.061 b	0.132 ± 0.057 bc	0.205 ± 0.056 a	0.101 ± 0.027 bcde	<0.001
GLA	18:3 n-6	0.024 ± 0.005 a	0.016 ± 0.004 c	0.016 ± 0.003 c	0.012 ± 0.003 c	0.029 ± 0.009 a	0.018 ± 0.005 bc	0.022 ± 0.006 ab	0.016 ± 0.002 c	<0.001
Arachidic	20:0	0.008 ± 0.002 cd	0.008 ± 0.002 cd	0.008 ± 0.001 cd	0.007 ± 0.001 d	0.014 ± 0.005 a	0.011 ± 0.002 ab	0.014 ± 0.002 a	0.009 ± 0.001 bc	<0.001
Eicosenoic	20:1 n-9	0.054 ± 0.006 cd	0.048 ± 0.009 de	0.044 ± 0.007 de	0.040 ± 0.003 e	0.083 ± 0.020 a	0.062 ± 0.009 bc	0.069 ± 0.009 ab	0.056 ± 0.006 cd	<0.001
Eicosedienoic	20:2 n-6	0.028 ± 0.007 c	0.020 ± 0.006 cd	0.020 ± 0.010 cd	0.010 ± 0.001 d	0.064 ± 0.037 a	0.035 ± 0.016 bc	0.054 ± 0.016 ab	0.025 ± 0.008 cd	<0.001
Eicosatrenoic	20:3 n-3	LOD	LOD	LOD	LOD	LOD	LOD	LOD	LOD	ND
DGLA	20:3 n-6	0.021 ± 0.006 bcd	0.013 ± 0.003 d	0.012 ± 0.004 d	0.008 ± 0.001 d	0.053 ± 0.028 a	0.030 ± 0.011 bc	0.034 ± 0.006 b	0.017 ± 0.003 cd	<0.001
Mead	20:9 n-9	0.006 ± 0.002 cd	0.005 ± 0.002 cd	0.004 ± 0.001 d	0.003 ± 0.001 d	0.016 ± 0.011 a	0.012 ± 0.005 ab	0.010 ± 0.003 bc	0.006 ± 0.001 cd	<0.001
Arachidonic	20:4 n-6	0.236 ± 0.049 a	0.159 ± 0.040 bc	0.197 ± 0.040 ab	0.139 ± 0.019 cd	0.241 ± 0.056 a	0.101 ± 0.029 d	0.156 ± 0.029 bc	0.105 ± 0.019 d	<0.001
EPA	20:5 n-3	0.007 ± 0.002 bc	0.006 ± 0.002 bc	0.004 ± 0.002 c	0.005 ± 0.001 c	0.017 ± 0.009 a	0.012 ± 0.007 ab	0.014 ± 0.006 a	0.007 ± 0.002 bc	<0.001
Behenic	22:00	LOD	LOD	LOD	LOD	LOD	LOD	LOD	LOD	ND
DTA	22:4 n-6	LOD	LOD	LOD	LOD	LOD	LOD	LOD	LOD	ND
DPA	22:5 n-3	0.169 ± 0.059 cd	0.114 ± 0.040 d	0.085 ± 0.033 d	0.080 ± 0.015 d	0.423 ± 0.232 a	0.267 ± 0.141 bc	0.316 ± 0.071 ab	0.155 ± 0.036 cd	<0.001
	22:5 n-6	0.119 ± 0.042 b	0.072 ± 0.020 bc	0.064 ± 0.015 bc	0.043 ± 0.008 c	0.244 ± 0.150 a	0.136 ± 0.037 b	0.137 ± 0.038 b	0.081 ± 0.025 bc	<0.001
DHA	22:6 n-3	0.199 ± 0.045 cd	0.114 ± 0.031 d	0.089 ± 0.023 d	0.074 ± 0.012 d	0.538 ± 0.275 a	0.272 ± 0.093 bc	0.406 ± 0.049 ab	0.168 ± 0.043 cd	<0.001

Lignoceric	24:0	LOD	LOD	LOD	LOD	LOD	LOD	LOD	LOD	ND
Total Cholesterol		0.809 ± 0.156 d	0.931 ± 0.138 cd	0.990 ± 0.108 bc	1.176 ± 0.099 a	1.208 ± 0.164 a	1.153 ± 0.142 ab	0.919 ± 0.112 cd	0.917 ± 0.149 cd	<0.001
Total SFA		6.157 ± 1.396 a	4.660 ± 0.623 bcd	5.159 ± 0.521 abc	4.264 ± 0.461 cd	5.433 ± 0.943 ab	3.712 ± 0.931 d	5.170 ± 0.817 abc	3.890 ± 0.649 d	<0.001
Total MUFA		9.532 ± 1.746 a	6.336 ± 0.612 bc	7.129 ± 0.761 b	6.401 ± 0.804 bc	7.406 ± 1.458 b	5.134 ± 1.579 c	7.322 ± 0.870 b	5.103 ± 0.818 c	<0.001
Total cis-MUFA		9.490 ± 1.739 a	6.300 ± 0.608 bc	7.096 ± 0.755 b	6.375 ± 0.802 bc	7.357 ± 1.441 b	5.089 ± 1.565 c	7.268 ± 0.861 b	5.066 ± 0.814 c	<0.001
total trans-MUFA		0.042 ± 0.009 abc	0.036 ± 0.006 bcd	0.033 ± 0.008 cd	0.026 ± 0.005 d	0.049 ± 0.021 ab	0.045 ± 0.015 abc	0.054 ± 0.011 a	0.037 ± 0.006 bcd	<0.001
Total PUFA		4.334 ± 1.141 a	2.639 ± 0.612 bc	3.609 ± 1.270 ab	2.343 ± 0.338 c	4.430 ± 1.259 a	2.499 ± 0.897 bc	4.042 ± 0.598 a	2.297 ± 0.375 c	<0.001
Total n-6		3.833 ± 1.063 a	2.296 ± 0.579 bc	3.343 ± 1.234 a	2.104 ± 0.328 c	3.285 ± 0.820 a	1.796 ± 0.643 c	3.088 ± 0.502 ab	1.850che ± 0.306 c	<0.001
Total n-3		0.516 ± 0.140 cd	0.354 ± 0.138 d	0.236 ± 0.066 d	0.234 ± 0.051 d	1.349 ± 0.661 a	0.651 ± 0.206 bc	0.968 ± 0.244 ab	0.421 ± 0.072 cd	<0.001
n-6:n-3 ratio		7.556 ± 1.759 b	6.402 ± 2.575 bc	13.924 ± 5.787 a	8.890 ± 0.926 b	2.660 ± 2.427 cd	2.607 ± 0.468 d	3.223 ± 0.587 d	4.203 ± 0.976 cd	<0.001
Total OCFA		0.055 ± 0.010 ab	0.045 ± 0.013 bc	0.045 ± 0.007 bc	0.037 ± 0.004 c	0.061 ± 0.013 a	0.040 ± 0.010 c	0.059 ± 0.008 a	0.041 ± 0.007 c	<0.001
Total OBCFA		3.768 ± 0.340 ab	4.111 ± 0.773 a	3.286 ± 0.145 c	3.580 ± 0.428 bc	3.294 ± 0.249 bc	3.322 ± 0.370 bc	3.329 ± 0.084 bc	3.117 ± 0.128 c	<0.001
Total FA		20.160 ± 3.931 a	13.774 ± 1.703 cde	16.025 ± 1.894 bcd	13.128 ± 1.482 de	17.436 ± 3.470 ab	11.485 ± 3.171 e	16.692 ± 1.939 bc	11.427 ± 1.741 e	<0.001

[1]Means ± standard deviation n = 24 eggs pooled into n = 12 replicates per month [2]Results of one-way ANOVA. a-e, Means within a row with different letters significantly differ $p < 0.05$. SFA, saturated fatty acids; MUFA, monounsaturated fatty acids, PUFA, polyunsaturated fatty acids; OCFA, odd-chain fatty acids; FA, fatty acids.
