# Supplementary material for: Seasonal variation in egg nutrient composition under a pasture-based layer hen system: Implications for sustainable agriculture
Source: PLoS One. 2025 Sep 25;20(9):e0332411. doi: 10.1371/journal.pone.0332411 (PMC12463277; doi:10.1371/journal.pone.0332411)
Supplement: S7 Table — (PDF) [file pone.0332411.s007.pdf]

Table S7. Egg yolk fatty acids by month (% of total fatty acids)[1]

Fatty Acid	Carbon Number	May	Jun	Jul	Aug	Sept	Oct	Nov	Dec	*p*-value[2]
Caprylic	8:0	LOD	LOD	LOD	LOD	LOD	LOD	LOD	LOD	ND
Capric	10:0	0.005 ± 0.002 c	0.010 ± 0.002 ab	0.010 ± 0.002 bc	0.012 ± 0.003 a	0.007 ± 0.004 bc	0.007 ± 0.001 bc	0.006 ± 0.001 c	0.008 ± 0.001 bc	<0.001
Undecanoic	11:0	0.001 ± 0.001 cd	0.004 ± 0.001 a	0.003 ± 0.002 ab	0.004 ± 0.001 a	0.002 ± 0.002 bc	0.001 ± 0.001 cd	0.001 ± 0.001 d	0.001 ± 0.001 d	<0.001
Lauric	12:0	0.002 ± 0.001 cd	0.003 ± 0.001 b	0.004 ± 0.001 ab	0.005 ± 0.001 a	0.002 ± 0.001 bc	0.002 ± 0.001 cd	0.002 ± 0.001 d	0.002 ± 0 .001cd	<0.001
Tridecanoic	13:0	0.009 ± 0.001 c	0.016 ± 0.002 a	0.014 ± 0.003 a	0.016 ± 0.005 a	0.011 ± 0.003 b	0.011 ± 0.003 bc	0.010 ± 0.001 bc	0.011 ± 0.001 bc	<0.001
Myristic	14:0	0.298 ± 0.066 c	0.442 ± 0.087 a	0.310 ± 0.056 c	0.354 ± 0.066 bc	0.323 ± 0.045 bc	0.360 ± 0.081 bc	0.398 ± 0.037 b	0.320 ± 0.029 c	<0.001
Myristoleic	14:1	0.047 ± 0.014 e	0.102 ± 0.039 a	0.047 ± 0.031 de	0.079 ± 0.023 abcd	0.076 ± 0.032 bcde	0.112 ± 0.037 ab	0.094 ± 0.008 abc	0.064 ± 0.016 cde	<0.001
Pentadecanoic	15:0	0.071 ± 0.011 b	0.080 ± 0.008 a	0.069 ± 0.006 b	0.076 ± 0.007 ab	0.090 ± 0.012 a	0.078 ± 0.017 ab	0.093 ± 0.013 a	0.080 ± 0.01 ab	<0.001
Palmitic	16:0	24.562 ± 1.931 b	26.844 ± 1.482 a	25.660 ± 2.528 ab	25.915 ± 1.182 ab	25.611 ± 2.946 ab	26.309 ± 1.942 ab	24.553 ± 2.531 ab	26.786 ± 1.685 ab	0.005
Palmiteladic	16:1 n-9t	0.044 ± 0.006 c	0.050 ± 0.024 c	0.046 ± 0.009 c	0.052 ± 0.014 bc	0.066 ± 0.012 ab	0.076 ± 0.012 a	0.077 ± 0.016 a	0.066 ± 0.016 ab	<0.001
Palmitoleic	16:1 n-7	0.738 ± 0.056 a	0.566 ± 0.093 bc	0.597 ± 0.089 b	0.449 ± 0.095 c	0.554 ± 0.159 b	0.567 ± 0.064 bc	0.561 ± 0.105 bc	0.652 ± 0.2 ab	<0.001
	16:1 n-9	2.465 ± 0.332 cd	3.421 ± 0.990 ab	2.232 ± 0.883 d	3.141 ± 0.491 abc	3.112 ± 1.054 bc	3.991 ± 0.974 a	3.518 ± 0.336 ab	3.136 ± 0.812 bc	<0.001
Heptadecanoic	17:0	0.192 ± 0.030 c	0.206 ± 0.025 bc	0.196 ± 0.012 c	0.190 ± 0.024 c	0.242 ± 0.045 ab	0.256 ± 0.056 a	0.244 ± 0.031 ab	0.260 ± 0.032 a	<0.001
c10-heptadecanoic	17:1	LOD	LOD	LOD	LOD	LOD	LOD	LOD	LOD	ND
Stearic	18:0	5.283 ± 0.487 c	6.123 ± 0.677 abc	6.829 ± 0.593 a	5.670 ± 1.047 bc	5.755 ± 0.686 c	5.668 ± 1.117 bc	5.348 ± 0.700 c	6.690 ± 0.888 ab	<0.001
Eladic	18:1 n-9t	0.171 ± 0.030 cd	0.218 ± 0.033 bc	0.163 ± 0.059 cd	0.142 ± 0.035 d	0.214 ± 0.087 bc	0.294 ± 0.086 a	0.239 ± 0.038 b	0.264 ± 0.050 ab	<0.001
Oleic	18:1 n-9	42.227 ± 2.610 ab	39.433 ± 2.089 bcd	40.328 ± 2.869 abc	43.580 ± 1.806 a	35.698 ± 3.388 e	36.008 ± 4.656 de	36.662 ± 4.279 de	37.911 ± 3.768 cde	<0.001
	18:1 n-11	1.611 ± 0.096 c	1.746 ± 0.314 bc	1.156 ± 0.315 d	1.380 ± 0.162 cd	2.184 ± 0.804 ab	2.406 ± 0.378 a	2.327 ± 0.298 a	2.044 ± 0.458 ab	<0.001
Linoleic	18:2 n-6	16.502 ± 2.137 ab	14.578 ± 2.938 bc	18.653 ± 5.979 a	14.180 ± 1.866 bc	15.217 ± 2.840 abc	12.469 ± 3.610 c	15.669 ± 2.515 abc	13.834 ± 1.627 bc	0.001
CLA	9c, 11t 18:2	0.081 ± 0.012 c	0.098 ± 0.011 c	0.073 ± 0.009 c	0.084 ± 0.015 c	0.125 ± 0.038 b	0.150 ± 0.026 a	0.126 ± 0.010 b	0.132 ± 0.017 ab	<0.001
	11t, 13c	0.045 ± 0.012 d	0.068 ± 0.008 bc	0.056 ± 0.005 cd	0.064 ± 0.016 bcd	0.076 ± 0.018 bc	0.090 ± 0.020 a	0.064 ± 0.009 cd	0.081 ± 0.010 ab	<0.001
	11t, 13t	0.155 ± 0.022 d	0.206 ± 0.022 cd	0.158 ± 0.023 d	0.182 ± 0.042 d	0.254 ± 0.058 bc	0.298 ± 0.057 a	0.236 ± 0.018 bc	0.268 ± 0.037 ab	<0.001
	t, t	0.045 ± 0.009 c	0.071 ± 0.013 ab	0.059 ± 0.005 bc	0.064 ± 0.017 abc	0.062 ± 0.022 abc	0.081 ± 0.018 a	0.059 ± 0.009 bc	0.078 ± 0.006 a	<0.001
ALA	18:3 n-3	0.589 ± 0.144 de	0.745 ± 0.147 cd	0.484 ± 0.105 e	0.598 ± 0.114 de	0.874 ± 0.353 bc	1.180 ± 0.249 a	1.264 ± 0.246 a	0.966 ± 0.107 b	<0.001
GLA	18:3 n-6	0.12 ± 0.016 bcd	0.118 ± 0.024 cd	0.098 ± 0.006 d	0.096 ± 0.017 d	0.150 ± 0.034 ab	0.152 ± 0.027 a	0.134 ± 0.033 abc	0.150 ± 0.029 abc	<0.001
Arachidic	20:0	0.042 ± 0.007 e	0.058 ± 0.007 cd	0.046 ± 0.006 de	0.050 ± 0.007 de	0.074 ± 0.018 bc	0.098 ± 0.020 a	0.076 ± 0.009 b	0.084 ± 0.008 ab	<0.001
Eicosenoic	20:1 n-9	0.274 ± 0.036 d	0.356 ± 0.040 cd	0.284 ± 0.020 d	0.306 ± 0.054 d	0.434 ± 0.069 b	0.546 ± 0.101 a	0.422 ± 0.033 bc	0.478 ± 0.056 ab	<0.001
Eicosedienoic	20:2 n-6	0.130 ± 0.039 b	0.144 ± 0.048 b	0.120 ± 0.053 b	0.076 ± 0.023 b	0.332 ± 0.174 a	0.306 ± 0.092 a	0.304 ± 0.061 a	0.225 ± 0.066 a	<0.001
Eicosatrenoic	20:3 n-3	LOD	LOD	LOD	LOD	LOD	LOD	LOD	LOD	ND
DGLA	20:3 n-6	0.100 ± 0.022 de	0.104 ± 0.020 de	0.074 ± 0.030 e	0.062 ± 0.010 e	0.334 ± 0.109 a	0.240 ± 0.045 ab	0.217 ± 0.049 bc	0.150 ± 0.027 cd	<0.001
Mead	20:9 n-9	0.030 ± 0.011 cd	0.035 ± 0.016 cd	0.027 ± 0.010 d	0.024 ± 0.009 d	0.086 ± 0.072 ab	0.103 ± 0.055 a	0.058 ± 0.020 bc	0.048 ± 0.018 cd	<0.001
Arachidonic	20:4 n-6	1.172 ± 0.167 b	1.180 ± 0.364 b	1.236 ± 0.171 ab	1.046 ± 0.163 bc	1.383 ± 0.333 a	0.934 ± 0.132 c	0.907 ± 0.187 c	0.889 ± 0.223 c	<0.001
EPA	20:5 n-3	0.031 ± 0.003 c	0.040 ± 0.010 c	0.026 ± 0.005 c	0.030 ± 0.008 c	0.098 ± 0.040 a	0.086 ± 0.007 a	0.074 ± 0.019 ab	0.056 ± 0.014 bc	<0.001
Behenic	22:00	LOD	LOD	LOD	LOD	LOD	LOD	LOD	LOD	ND
DTA	22:4 n-6	LOD	LOD	LOD	LOD	LOD	LOD	LOD	LOD	ND
DPA	22:5 n-3	0.869 ± 0.336 cd	0.876 ± 0.311 cd	0.487 ± 0.218 d	0.604 ± 0.165 d	2.620 ± 1.262 a	1.954 ± 0.778 a	1.771 ± 0.324 ab	1.318 ± 0.428 bc	<0.001
	22:5 n-6	0.638 ± 0.204 bcd	0.473 ± 0.122 bcd	0.408 ± 0.061 cd	0.320 ± 0.038 d	1.412 ± 0.788 a	1.194 ± 0.305 a	0.748 ± 0.336 b	0.724 ± 0.250 bc	<0.001
DHA	22:6 n-3	0.984 ± 0.179 bc	0.879 ± 0.267 bc	0.548 ± 0.086 c	0.567 ± 0.064 c	3.574 ± 1.401 a	2.173 ± 0.437 a	2.455 ± 0.188 a	1.482 ± 0.352 b	<0.001
Lignoceric	24:0	LOD	LOD	LOD	LOD	LOD	LOD	LOD	LOD	ND
C14:0-*iso*	14:0	LOD	LOD	LOD	LOD	LOD	LOD	LOD	LOD	ND
C15:0-*iso*	15:0	0.019 ± 0.005 bc	0.042 ± 0.019 a	0.016 ± 0.004 c	0.023 ± 0.006 bc	0.019 ± 0.003 bc	0.022 ± 0.005 b	0.017 ± 0.003 c	0.020 ± 0.004 bc	<0.001
C15:0-*anteiso*	15:0	0.010 ± 0.002 c	0.015 ± 0.002 ab	0.012 ± 0.003 bc	0.015 ± 0.004 ab	0.014 ± 0.005 ab	0.017 ± 0.004 a	0.012 ± 0.002 bc	0.016 ± 0.002 a	<0.001
C16:0-*iso*	16:0	0.048 ± 0.010 c	0.076 ± 0.013 ab	0.064 ± 0.009 bc	0.077 ± 0.016 abc	0.074 ± 0.027 abc	0.091 ± 0.022 a	0.063 ± 0.010 bc	0.087 ± 0.010 a	<0.001
C17:0-*iso*	17:0	0.064 ± 0.010 d	0.100 ± 0.030 abc	0.079 ± 0.012 cd	0.095 ± 0.022 abcd	0.092 ± 0.027 bcd	0.112 ± 0.025 a	0.079 ± 0.012 cd	0.107 ± 0.015 ab	<0.001

C17:0-*anteiso*	17:0	0.068 ± 0.014 c	0.107 ± 0.019 ab	0.086 ± 0.013 bc	0.108 ± 0.028 ab	0.100 ± 0.025 abc	0.116 ± 0.028 a	0.084 ± 0.014 bc	0.110 ± 0.013 a	<0.001
C18:0-*iso*	18:0	0.138 ± 0.034 c	0.212 ± 0.040 bc	0.186 ± 0.024 c	0.211 ± 0.045 bc	0.199 ± 0.075 bc	0.272 ± 0.065 a	0.186 ± 0.027 c	0.257 ± 0.029 ab	<0.001
C18:0-*anteiso*	18:0	0.139 ± 0.035 c	0.211 ± 0.039 bc	0.186 ± 0.023 c	0.211 ± 0.044 bc	0.199 ± 0.075 bc	0.271 ± 0.065 a	0.184 ± 0.026 c	0.257 ± 0.028 ab	<0.001
Total SFA		30.932 ± 2.591 b	33.948 ± 2.392 a	32.904 ± 2.720 ab	32.740 ± 1.931 ab	31.767 ± 3.521 ab	32.701 ± 3.615 ab	30.889 ± 2.907 b	34.496 ± 1.425 a	<0.001
Total MUFA		47.489 ± 2.413 ab	46.119 ± 2.659 abc	45.311 ± 3.698 bc	49.147 ± 2.236 a	42.484 ± 1.868 c	44.094 ± 4.779 bc	44.136 ± 4.759 bc	45.288 ± 3.592 bc	<0.001
Total cis-MUFA		47.286 ± 2.343 ab	45.842 ± 2.605 abc	45.124 ± 3.654 bc	48.974 ± 2.261 a	42.181 ± 1.941 c	43.761 ± 4.834 bc	43.792 ± 4.711 bc	44.980 ± 3.638 bc	<0.001
total trans-MUFA		0.204 ± 0.041 cd	0.265 ± 0.042 bcd	0.210 ± 0.068 cd	0.192 ± 0.043 d	0.284 ± 0.079 bc	0.372 ± 0.112 a	0.316 ± 0.065 ab	0.326 ± 0.054 ab	<0.001
Total PUFA		20.640 ± 2.203 abcd	19.166 ± 2.089 cd	21.882 ± 5.833 abc	17.746 ± 1.839 d	25.702 ± 2.843 a	21.752 ± 4.498 abcd	23.990 ± 2.617 ab	19.967 ± 2.552 bcd	<0.001
Total n-6		18.446 ± 1.972 ab	16.424 ± 2.322 b	20.548 ± 6.173 a	15.875 ± 1.979 b	18.384 ± 2.209 ab	15.375 ± 3.820 b	18.375 ± 2.948 ab	16.000 ± 1.811 b	<0.001
Total n-3		2.448 ± 0.210 bc	2.598 ± 0.794 bc	1.568 ± 0.389 c	1.802 ± 0.280 c	7.361 ± 2.662 a	5.401 ± 1.307 a	5.668 ± 0.417 a	3.841 ± 0.722 b	<0.001
n-6:n-3 ratio		7.556 ± 1.759 b	6.402 ± 2.575 bc	13.924 ± 5.787 a	8.890 ± 0.926 b	2.660 ± 2.427 cd	2.607 ± 0.468 d	3.223 ± 0.587 d	4.203 ± 0.976 cd	<0.001
Total CLA		0.328 ± 0.050 e	0.442 ± 0.036 cde	0.345 ± 0.045 de	0.390 ± 0.091 cde	0.488 ± 0.143 bcd	0.619 ± 0.120 a	0.486 ± 0.037 bc	0.557 ± 0.078 ab	<0.001
Total OCFA		0.162 ± 0.023 d	0.264 ± 0.070 ab	0.197 ± 0.034 cd	0.248 ± 0.061 abc	0.226 ± 0.058 bcd	0.266 ± 0.056 a	0.192 ± 0.032 cd	0.253 ± 0.033 ab	<0.001
Total OBCFA		0.273 ± 0.046 b	0.304 ± 0.032 ab	0.285 ± 0.018 b	0.286 ± 0.020 b	0.346 ± 0.048 a	0.338 ± 0.074 a	0.344 ± 0.048 a	0.348 ± 0.038 a	<0.001
Total BCFA		0.347 ± 0.065 d	0.531 ± 0.108 abc	0.442 ± 0.068 cd	0.550 ± 0.113 abc	0.469 ± 0.162 bcd	0.629 ± 0.151 a	0.440 ± 0.069 cd	0.597 ± 0.075 ab	<0.001
Total *iso*BCFA		0.270 ± 0.050 d	0.412 ± 0.091 abc	0.343 ± 0.050 cd	0.422 ± 0.084 abcd	0.363 ± 0.143 bcd	0.496 ± 0.120 a	0.345 ± 0.053 cd	0.471 ± 0.058 ab	<0.001
Total *anteiso*BCFA		0.078 ± 0.015 c	0.121 ± 0.019 ab	0.098 ± 0.017 bc	0.123 ± 0.031 ab	0.114 ± 0.029 ab	0.134 ± 0.032 a	0.096 ± 0.016 bc	0.126 ± 0.014 a	<0.001

[1]Means ± standard deviation n = 24 eggs pooled into n = 12 replicates per month [2]Results of one-way ANOVA. a-e, Means within a row with different letters significantly differ $p < 0.05$. SFA, saturated fatty acids; MUFA, monounsaturated fatty acids, PUFA, polyunsaturated fatty acids; CLA, conjugated linoleic acid; OCFA, odd-chain fatty acids; OCBFA, odd-chain branched fatty acids; BCFA, branch-chain fatty acids; FA, fatty acids.
