# Supplementary material for: Seasonal variation in egg nutrient composition under a pasture-based layer hen system: Implications for sustainable agriculture
Source: PLoS One. 2025 Sep 25;20(9):e0332411. doi: 10.1371/journal.pone.0332411 (PMC12463277; doi:10.1371/journal.pone.0332411)
Supplement: S8 Table — (PDF) [file pone.0332411.s008.pdf]

Table S8. Yolk mineral profile of the egg yolks by month[1]

Parameter	May	Jun	Jul	Aug	Sept	Oct	Nov	Dec	*p*-value[2]
Iron (ug/g FW)	69.76 ± 7.30 abc	70.66 ± 5.39 ab	64.73 ± 6.10 bc	63.49 ± 5.37 c	71.90 ± 6.58 a	66.44 ± 5.02 abc	66.09 ± 3.51 abc	63.60 ± 4.00 c	<0.001
Zinc (ug/g FW)	34.26 ± 1.68	34.80 ± 1.44	35.46 ± 1.27	34.61 ± 1.72	33.37 ± 2.27	34.77 ± 1.75	33.70 ± 1.55	34.07 ± 1.47	0.073
Copper (ug/g FW)	1.89 ± 0.14 bc	1.83 ± 0.11 c	2.02 ± 0.13 ab	1.94 ± 0.17 abc	2.09 ± 0.13 a	2.09 ± 0.10 a	1.95 ± 0.09 abc	1.82 ± 0.13 c	<0.001
Manganese (ug/g FW)	0.86 ± 0.20 a	0.70 ± 0.11 ab	0.70 ± 0.08 ab	0.58 ± 0.13 b	0.64 ± 0.17 b	0.73 ± 0.13 ab	0.87 ± 0.14 a	0.69 ± 0.09 b	<0.001
Molybdenum (ug/g FW)	0.19 ± 0.07 a	0.17 ± 0.03 ab	0.12 ± 0.04 b	0.11 ± 0.03 b	0.20 ± 0.07 a	0.16 ± 0.03 ab	0.13 ± 0.02 b	0.16 ± 0.03 ab	<0.001
Selenium (ug/g FW)	0.67 ± 0.13 c	0.61 ± 0.09 c	0.57 ± 0.15 c	0.71 ± 0.14 bc	1.00 ± 0.15 a	0.89 ± 0.16 a	0.87 ± 0.13 ab	0.87 ± 0.14 ab	<0.001
Calcium (ug/g FW)	1227.07 ± 69.38	1188.78 ± 58.28	1247.08 ± 65.68	1181.52 ± 68.76	1171.15 ± 74.45	1197.93 ± 67.25	1199.52 ± 74.92	1177.54 ± 60.02	0.101
Magnesium (ug/g FW)	115.05 ± 6.65 bc	116.36 ± 6.50 b	126.13 ± 6.63 a	120.11 ± 8.41 ab	107.66 ± 7.67 c	115.36 ± 5.27 bc	116.64 ± 5.68 b	117.18 ± 7.25 b	<0.001
Potassium (ug/g FW)	1195.41 ± 69.45 ab	1191.64 ± 59.40 ab	1146.69 ± 120.20 b	1195.46 ± 67.51 ab	1264.26 ± 88.64 a	1248.51 ± 61.92 ab	1155.18 ± 40.16 b	1207.58 ± 118.01 ab	0.009
Phosphorus (ug/g FW)	5444.32 ± 106.85 bcd	5415.41 ± 39.19 cd	5574.79 ± 66.39 a	5511.99 ± 56.71 ab	5386.93 ± 106.02 d	5522.30 ± 51.21 ab	5507.46 ± 78.10 abc	5552.88 ± 69.01 a	<0.001
Sulfur (ug/g FW)	1435.44 ± 40.92	1409.58 ± 44.86	1453.27 ± 39.97	1465.40 ± 44.33	1412.41 ± 42.94	1441.20 ± 50.34	1422.17 ± 60.32	1441.75 ± 41.34	0.046
Sodium (ug/g FW)	489.01 ± 26.20 abc	470.05 ± 24.70 bc	481.38 ± 146.93 abc	411.69 ± 206.77 c	530.85 ± 38.14 abc	561.95 ± 26.09 ab	579.69 ± 33.69 ab	605.72 ± 94.76 a	<0.001
Aluminum (ug/g FW)	1.12 ± 0.25 cd	1.18 ± 0.18 bcd	1.31 ± 0.39 bcd	1.29 ± 0.24 bcd	1.49 ± 0.25 abc	1.67 ± 0.24 ab	1.88 ± 0.91 a	0.84 ± 0.18 d	<0.001

[1]Means ± standard deviation n = 24 eggs pooled into n = 12 replicates per month [2]Results of one-way ANOVA. a-e, Means within a row with different letters significantly differ $p < 0.05$. FW, fresh weight.
