# Supplementary material for: Seasonal variation in egg nutrient composition under a pasture-based layer hen system: Implications for sustainable agriculture
Source: PLoS One. 2025 Sep 25;20(9):e0332411. doi: 10.1371/journal.pone.0332411 (PMC12463277; doi:10.1371/journal.pone.0332411)
Supplement: S9 Table — (PDF) [file pone.0332411.s009.pdf]

Supplementary Table 9. Yolk sPLS-DA Loadings Plot Values[1]

Parameter	Component 1	Component 2
**Total n-3**	-0.449	0.000
**C22:6 n-3**	-0.439	-0.001
**C22:5 n-3**	-0.399	0.000
**C18:3 n-3**	-0.392	0.216
**n-6:n-3 ratio**	0.363	-0.235
**Vitamin E**	-0.363	-0.308
**Total MUFA**	0.123	0.000
**Total PUFA**	-0.109	-0.083
**Vitamin A**	-0.039	-0.742
**C20:5 n-3**	-0.032	0.085
**T. Carotenoids**	0.000	0.000
**Beta-Carotene**	0.000	0.000
**Total n-6**	0.000	-0.108
**T. Cholesterol**	0.000	-0.465
**Total SFA**	0.000	0.104
**T. Phenolics**	0.000	0.000

[1] sparse Partial Least Squares Discriminant Analysis (sPLS-DA) loadings for various yolk parameters across two principal components (PC1 and PC2). T. Cholesterol, total cholesterol, T. Carotenoids, total carotenoids, T. Phenolics; total phenolics, C18:3 n-3; ALA, C20:5 n-3; EPA, C22:5 n-3; DPA n-3, C22:6 n-3; DHA, SFA; saturated fatty acids, MUFA; monounsaturated fatty acids, PUFA; polyunsaturated fatty acids, n-6; omega-6 fatty acid, n-3; omega-3 fatty acid
