# Supplementary material for: Seasonal variation in egg nutrient composition under a pasture-based layer hen system: Implications for sustainable agriculture
Source: PLoS One. 2025 Sep 25;20(9):e0332411. doi: 10.1371/journal.pone.0332411 (PMC12463277; doi:10.1371/journal.pone.0332411)
Supplement: S10 Table — (PDF) [file pone.0332411.s010.pdf]

Supplementary Table 10. Forage and Feed sPLS-DA Loadings Plot Values[1]

Parameter	Component 1	Component 2
**Total n-6**	0.477	0.000
**n-6:n-3 ratio**	0.460	0.000
**C20:5 n-3**	0.413	0.000
**C22:6 n-3**	0.400	0.000
**C22:5 n-3**	0.321	0.000
**C18:3 n-3**	-0.251	-0.175
**Total n-3**	-0.217	-0.198
**Chlorophyll A**	-0.091	-0.205
**Total MUFA**	0.073	0.258
**Chlorophyll B**	-0.065	-0.203
**Total PUFA**	0.000	-0.555
**Total SFA**	0.000	0.468
**T. Phenolics**	0.000	-0.361
**Vitamin E**	0.000	0.253
**T. Carotenoids**	0.000	-0.243

[1]**sparse Partial Least Squares Discriminant Analysis (sPLS-DA)** loadings for various forage parameters across two principal components.C18:3 n-3, alpha-linolenic acid; C20:5 n-3, eicosapentaenoic acid (EPA); C22:5 n-3, docosapentaenoic acid (DPA); C22:6 n-3, docosahexaenoic acid (DHA); SFA, saturated fatty acids; MUFA, monounsaturated fatty acids; PUFA, polyunsaturated fatty acids; n-6, omega-6 fatty acids; n-3, omega-3 fatty acids; n-6:n-3 ratio, ratio of omega-6 to omega-3 fatty acids; Vitamin E, tocopherol; T. Carotenoids, total carotenoids; T. Phenolics, total phenolics.
